# Regulatory elements in vectors containing the ctEF-1α first intron and double enhancers for an efficient recombinant protein expression system

**DOI:** 10.1038/s41598-018-33500-0

**Published:** 2018-10-18

**Authors:** Chi-Pin Lee, Albert Min-Shan Ko, Shang-Lun Chiang, Chi-Yu Lu, Eing-Mei Tsai, Ying-Chin Ko

**Affiliations:** 1 Environment-Omics-Diseases Research Center, China Medical University Hospital, China Medical University, Taichung, 40402 Taiwan; 2 Key Laboratory of Vertebrate Evolution and Human Origins of Chinese Academy of Sciences, IVPP, CAS, Beijing, 100044 China; 3 Department of Health Risk Management, College of Public Health, China Medical University, Taichung, 40402 Taiwan; 4 Department of Biochemistry, College of Medicine, Kaohsiung Medical University, Kaohsiung, 80708 Taiwan; 5 Graduate Institute of Medicine, College of Medicine, Kaohsiung Medical University, Kaohsiung, 80708 Taiwan

## Abstract

To establish a stable and scalable transient protein production system, we modified the EF-1 first intron size and verified the order of two recombinant enhancers downstream of the SV40 polyA sequence. This new vector was named pHH-Gemini (pHH-GM1) and was used to express alpha kinase 1 (*ALPK1*) and various other proteins, NLRP3, F-actin, Camodulin, PP2A, URAT1, Rab11a and myosin IIA. The results showed that, compared with six commercial plasmids, pHH-GM1 significantly enhanced His-HA-ALPK1 expression in a western blot analysis of transfected HEK293T cells. The expression of various other genes was also successful using the pHH-GM1 vector. In addition, we inserted turbo green florescence protein (tGFP) into the pHH-GM1 vector, and an improvement in fluorescence intensity was observed after transient transfection of HEK293T cells. For large-scale production, protein production was tested by standard supplementation with one volume of medium, and volumetric yields of 2 and 2.3 mg/L were achieved with pHH-GM1-ALPK1 in HEK293-F and CHO-S cells, respectively. We found that cell viability was more than 70% 11 days after cells were transfected with the pHH-GM1 vector. The pHH-GM1 vector with the ctEF-1α first intron and double enhancers, Simian virus 40 and Cytomegalovirus (SV40 and CMV) is an efficient CMV promoter-based gene expression system that can potentially be applied to study genes of interest and improve protein production.

## Introduction

While bacteria are widely used to express recombinant proteins (r-proteins) due to their ease of use, high yield, and low cost, they lack eukaryotic post-translational modification systems^1^, which heterologous genes with abundant codons, rarely used in *E. coli*, may not be efficiently expressed in *E. coli* and may lead to translation error^2^, compromised protein function, improper protein folding, or protein aggregation into inclusion bodies^3,4^. Thus, transfer of foreign genes into mammalian cells also needs to be considered and can be achieved using plasmid-based or viral-mediated (e.g., adenovirus, vaccinia virus, retrovirus, baculovirus) methods. Recombinant proteins expression in mammalian cells has become an essential process in biopharmaceutical industries due to the ability of mammalian cells to express biologically active proteins. The appropriate choice of a mammalian expression system is likely to ensure proper protein folding and authentic post-translational modifications^5^. Transfection of mammalian cells is achieved either through stable gene expression (SGE) or transient gene expression (TGE). High mammalian protein production levels using recombinant systems is important for preclinical, biochemical, biophysical, and drug discovery studies performed by numerous scientific groups and pharmaceutical companies. Foreign gene expression efficiency is dependent on several factors^6^, such as the vector promoter and human elongation factor one alpha (*EF-1α*), which is abundantly found in most tissues and has been shown to be a strong promoter^7,8^. Insertion of the first intron of human *EF-1α* significantly improved the gene expression level in many cell lines, and combining the murine cytomegalovirus promoter with the human EF-1α first intron (MCMV/EF-I) induced 4.3- to 65.5-fold higher expression than the human cytomegalovirus promoter in a variety of normal and cancer cells^9^. The first intron of EF-1α contains several transcription factors Spl and Apl elements, which seem to have additive effects on its promoter activity^10^. These results showed that both the 5′ flanking region and the first intron of the EF-lα gene are essential for its promoter activity. Additionally, the transcriptional elements of human telomerase reverse transcriptase (hTERT), simian virus 40 (SV40) and cytomegalovirus (CMV) triple enhancers interact individually or synergistically with the CMV promoter, which further improves and enhances the transgene expression capacity of mammalian cells^8^. However, a variety of enhancer sequences can also be used to modify and regulate gene expression^11,12^. In this study, to identify an efficient expression system that can induce potent expression of various genes, including NLRP3 (inflammasome), ALPK1 (kinase protein), F-actin (motor protein), calmodulin, PP2A (phosphatase), URAT1 (membrane protein), Rab11a (cellular trafficking protein), and myosin IIA (cytoskeleton protein), we systematically inserted various combinations of the C-terminal EF-1α first intron (ctEF-1α) and two translational enhancers (SV40 and CMV) downstream of the SV40 polyA sequence in a CMV promoter-driven gene expression cassette.

## Results

### The SV40 and CMV double enhancers significantly enhances protein level in mammalian cells

**Figure 1:**
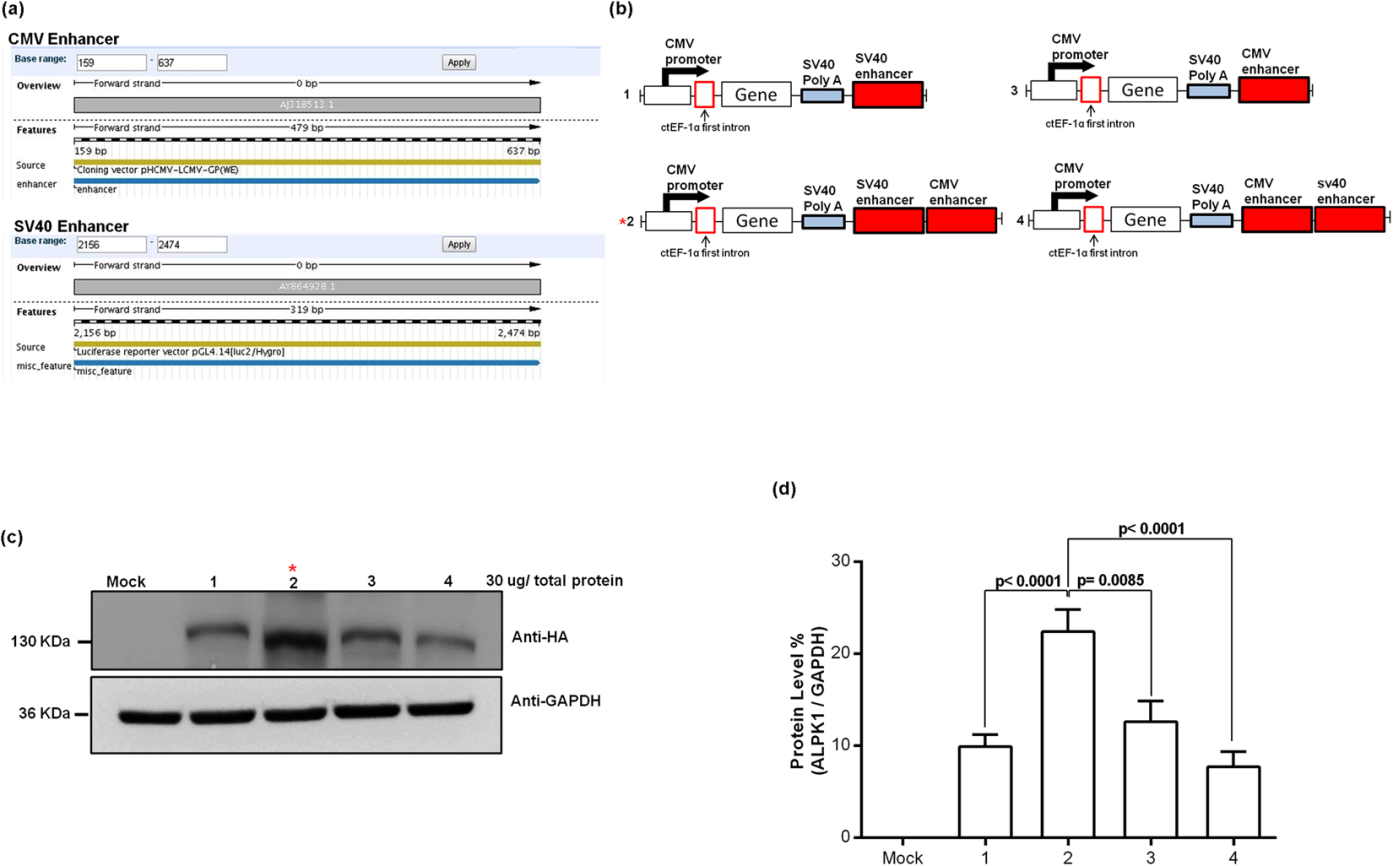
(**a**) The orientation of the CMV and SV40 enhancers: sequence map depicting cloning site and location on respective pHCMV-LCMV-GP and pGL4 vectors from the NCBI database. (**b**) Schematic representation of the differences pHH-ALPK1 in the single vs. dual translational enhancer sequences (CMV and SV40) downstream of the SV40 polyA sequence that was constructed *in vitro* by inserting 3.7 kb human ALPK1 cDNA excised from pFN21K-Halo-ALPK1 at the *Asis*I and *Pme*I sites. The whole constructs also inserted the human ctEF-1first intron (GenBank no: LT727183.1) behind the CMV promote. (**c**) Expression of ALPK1 protein in HEK293T cells determined by western blot analysis. (**d**) ALPK1 protein expression relative to that of GAPDH in (**c**) HEK293T cells; *P* = *0.089*, construct 1 vs. construct 3; *P* <* 0.0001*, construct 2 vs. construct 4. These data are expressed as the mean ± SD (n = 4). *P < *0.001*, construct 2 vs. constructs 1, 3 and 4. One-way analysis of variance and Wilcoxon signed-rank test were conducted to examine in relation between vectors and different protein levels.

We subcloned CMV and SV40 enhancers fragment from pHCMV-LCMV-GP and pGL4 vectors, respectively^8^, with information on sequence and position based on the NCBI database (Fig. 1a). Figure 1b shows that in a comparison between single and double enhancers, the optimal result was obtained using SV40 followed by CMV enhancer sequences. Figure 1c shows the apparent molecular weight of HA-ALPK1 (135 KDa), the lane 3 (construct 2) protein is expressed at approximate 1–1.5 fold higher level than other constructs (lane 2, 4 and 5). The comparison between construct 1 and 3, the results was shown expressing efficiency, CMV enhancer higher than SV40 enhancer (*p* = *0*.*089*). Surprisingly, the efficiency of protein expression, the SV40 followed by CMV enhancer (construct 2), but higher than the CMV followed by SV40 (construct 4) (*p* < *0*.*0001*). The comparison between construct 1, 2 and 3, the results were shown expressing efficiency, SV40 followed by CMV (construct 2) enhancer highest than CMV enhancer (construct 3) (*p* = *0*.*0085*) and SV40 enhancer (construct 1) (*p* < *0*.*0001*). Totally, the result shown double enhancers was superior single enhancer but dependent on order of enhancer elements. Moreover, the results also indicated that altering the order of the CMV and SV40 enhancers downstream of the SV40 polyA sequence changes the protein expression induced by this construct (Fig. 1d).

### The ctEF-1α element of vector enhanced transcription and translation

**Figure 2:**
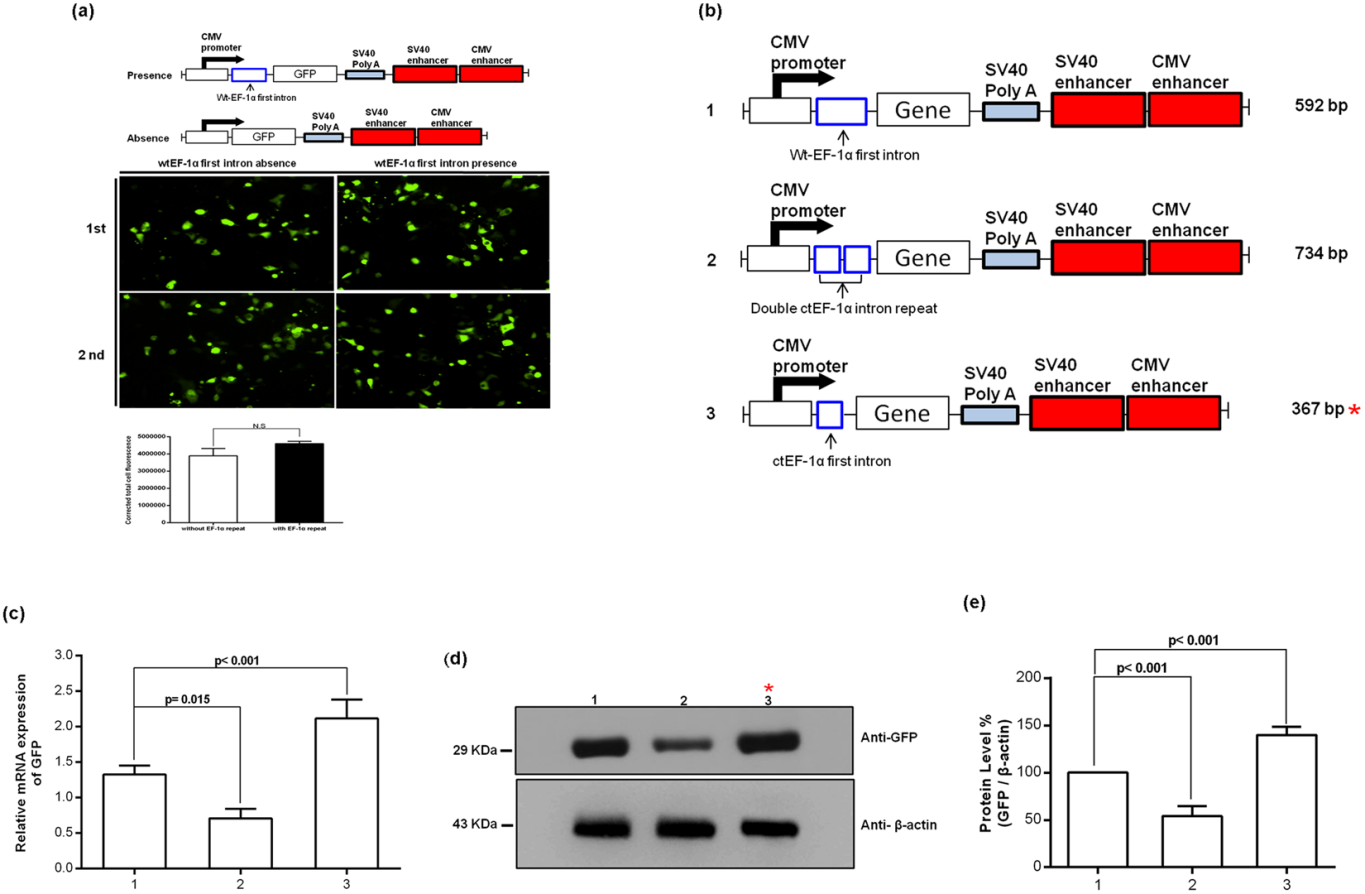
Construction of cassettes with/without the wild-type (wt) EF-1α first intron containing two translational enhancer sequences (CMV and SV40) downstream of SV40 polyA. (**a**) GFP expression after transfection of HEK293T cells with the two indicated constructs that included two enhancers and the presence or absence of the wtEF-1α first intron cassette. Protein expression analyses using fluorescence microscopy and quantification of the fluorescence image density. (**b**) Schematic representation of wt, double C-terminal (ct) and ct only EF-1α first intron cassettes with two translational enhancer sequences downstream of SV40 polyA. (**c**) GFP mRNA levels in HEK293T cells assessed using a real-time PCR assay. The gene expression levels, normalized to β-actin expression, are expressed as relative change, setting the values of the duodenum as one. The results are presented as the mean ± SD (n = 3 experiments). (**d**) Immunoblot analysis of GFP protein levels. (**e**) The immunoblot bands were quantified by densitometry analysis, and the β-actin ratios were calculated.

Figure 2a shows the effect of the presence of the wild-type (wt) EF-1α first intron in the promoter, which results in a minor increase in green fluorescent protein (GFP) protein expression, but the increase is not significant when compared with the absence of the intron. In Fig. 2b, the comparison of different EF-1α first intron sequences in the vector backbone containing two enhancers is shown. The ctEF-1α (C-terminal EF-1α) appeared to generate higher mRNA levels compared with wt and double ctEF-1α repeats. The highest protein expression was obtained with the ctEF-1α, and a further second inclusion reduced the signal. The constructs included either the wtEF-1α first intron, the ctEF-1α, or double ctEF-1α repeats downstream of the CMV promoter (Fig. 2c). The protein levels are also consistent with increased RNA levels (Fig. 2d). The GFP levels were quantified by β-actin using a western blot. GFP levels was higher in ctEF-1α than in wtEF-1α (*p* < *0*.*001*), but GFP was less in double ctEF-1α than in wtEF-1 (*p* < *0*.*001*; Fig. 2e). The result shown only single ctEF-1α driving GFP expression is best, but double is ctEF-1α worse rather than better. The new vector, which exhibited the most efficient gene expression ability, was named pHH-Gemini (pHH-GM1).

### The pHH-GM1 vector successful expresses protein in the different fraction

**Figure 3:**
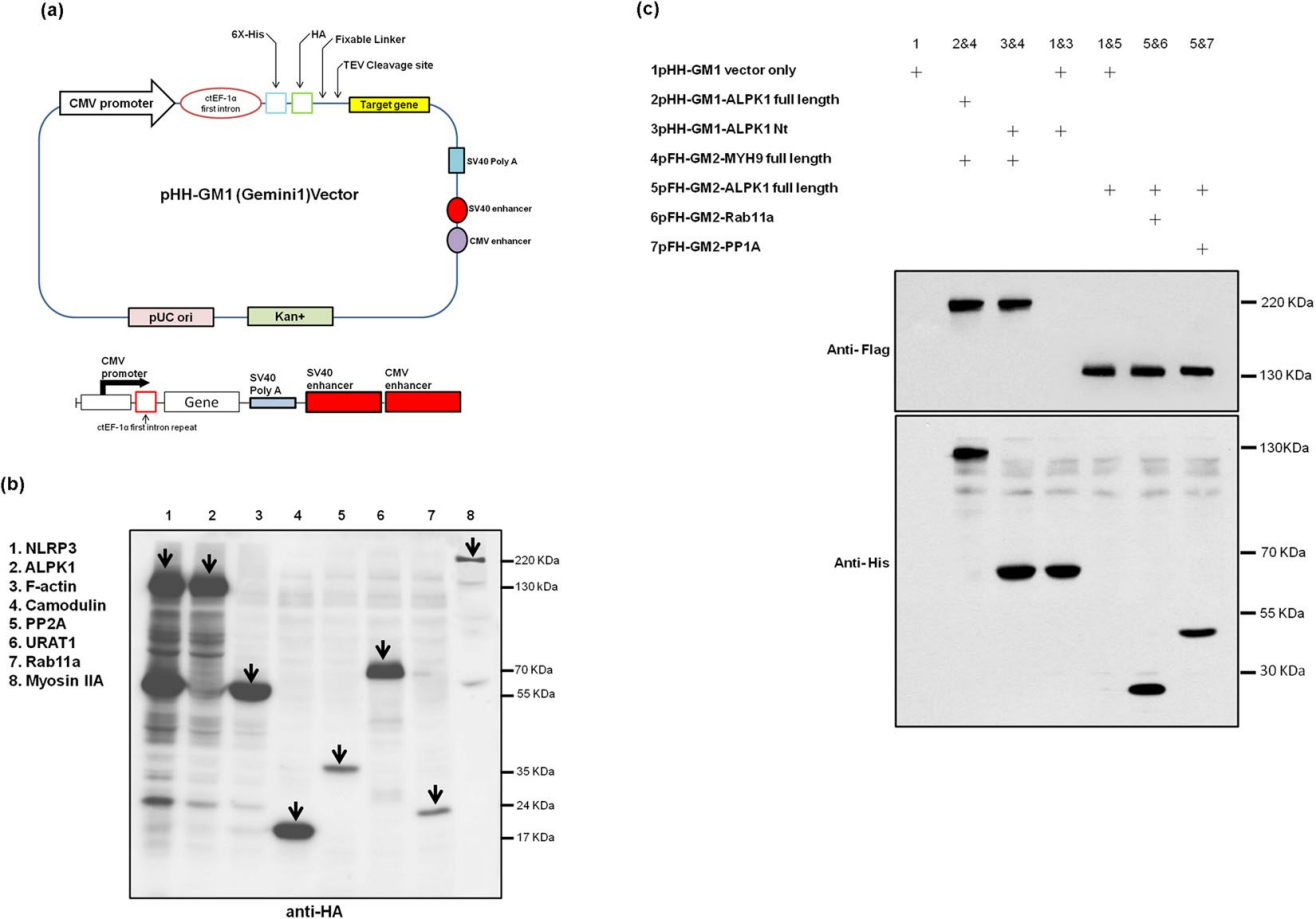
(**a**) Schematic diagram of the conventional gene expression system and the pHH-GM1 system. In the GM1 (Gemini) system, the C-terminal elongation factor 1α first intron (ctEF-1α) and double translational enhancer sequences (SV40 and CMV) were inserted downstream of the SV40 polyA sequence. (**b**) In this study, to identify an efficient expression system that can induce potent expression of various genes by western blotting using HA antibody, including NLRP3 (inflammasome), ALPK1 (kinase protein), F-actin (motor protein), calmodulin, PP2A (phosphatase), URAT1 (membrane protein), Rab11a (cellular trafficking protein), and myosin IIA (cytoskeleton protein), we systematically inserted various combinations of ctEF-1α, and double translational enhancers (SV40 and CMV) downstream of the SV40 polyA sequence in a CMV promoter-driven gene expression cassette. Arrows indicate protein bands unique to the variety HA-tagged fusion proteins. (**c**) All pHH-GM1 and pFH-GM2 vectors compatible test, randomly selected two constructs transfection to HEK293T. Successful recombinant protein expression after transient co-transfection of HEK293T cells with multiple separate plasmids carrying individual genes: pHH-GM1, tagged with His and HA, and pHF-GM2, tagged with His and FLAG.

The pHH-GM1 contains the ctEF-1α inserted downstream of the CMV promoter and double translational enhancers (SV40 and CMV) inserted downstream of the SV40 polyA sequence (Fig. 3a). In addition, vectors for the N-terminal epitope tags (6X-histidine and human influenza haemagglutinin (HA) with flexible linkers (Supplemental Fig. 3) can be applied in protein interaction and purification assays. pFH-GM2 construct component similar to pHH-GM1, only in N-terminal original HA epitope tag replace by FLAG (DYKDDDDK epitope). Figure 3b shows that various proteins (NLRP3, ALPK1, F-actin, calmodulin, PP2A, URAT1, Rab11a, and myosin IIA) can be produced with the pHH-GM1 vector. The ALPK1 X-ray crystallography and structural biology of work are not completed yet. The most important reason for ALPK1 protein expression difficultly, especially expressed in a bacterial system caused inclusion bodies formation.

### No interference effects for pHH-GM1 and pFH-GM2 with each other

Among these genes, ALPK1 was selected for further testing. When cells were transfected with plasmids having promoters, particularly strong promoters like CMV, there are enough DNA in many of the cells so that the promoters start competing with one another. As a result, we cotransfected HEK293T cells with multiple plasmids to test whether the two promoters interfered with each other through the Sp1-mediated CMV promoter, indicating a mechanism of competitive binding/inhibition. The results indicated that the two vectors using the same promoter with different tags (His and FLAG) do not interfere with each other and simultaneously induced expression of two different proteins (Fig. 3c). Generally, we expected one or both of the protein expressions might decrease due to interference with each other, however we did not observe any effect in band size from our test.

### The pHH-GM1 system improved and increased protein level in mammalian cells

**Figure 4:**
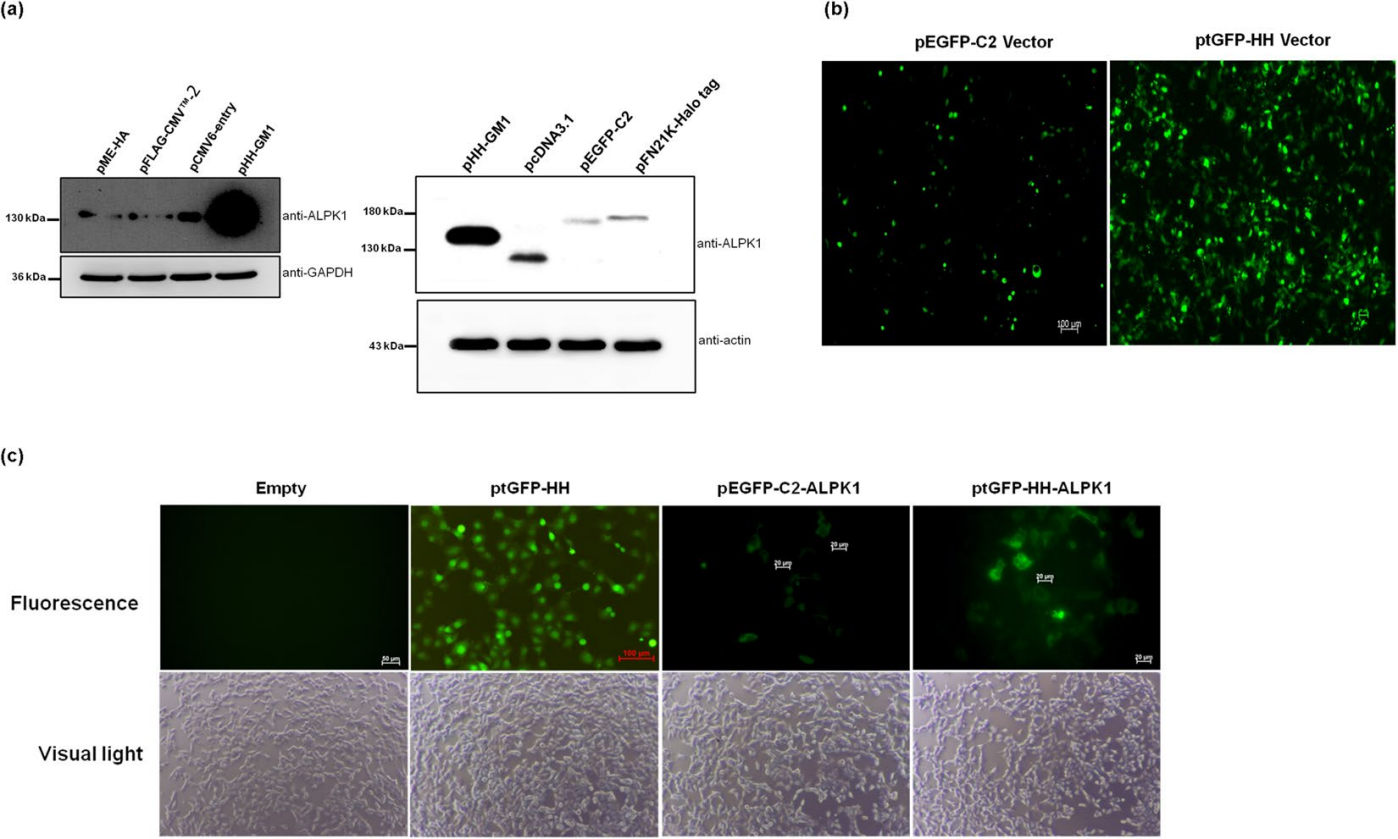
(**a**) pHH-GM1 and different commercial vectors (pME-HA, pFLAG-CMV-2, pCMV6-entry, pcDNA3.1, pEGFP-C2 and pFN21K-Halo-tag) were used for (**a**) comparing ALPK1 protein levels in HEK293T cells. (**b**) HEK293T cells transiently transfected with plasmid ptGFP-HH or pEGFP-C2 for GFP expression. Both DNAs harbour an identical gene expression cassette encoding GFP and were transfected using equivalent plasmid DNA. Images were taken at 24 h post-transfection. Magnification, 10x objective (with digitally enlarged insert). (**c**) HEK293T cells were grown to ~90% confluency in 6-well plate. GFP expression detected under a fluorescence microscope. The vectors carried the GFP gene; thus, a fluorescence microscope was used to detect GFP expression in HEK293T cells transfected with ptGFP-HH or pEGFP-C2 24 h after transfection. GFP expression in HEK293T cells transfected with ptGFP-HH was observed under a fluorescence or light microscope (magnification, ×10) (left 2, upper and lower). ptGFP-HH or pEGFP-C2 vectors carrying the GFP-ALPK1 fusion gene were transfected into HEK293T cells and observed at 24 h under a fluorescence or light microscope (magnification, ×20) (right 3 and 4, upper and lower).

Figure 4a shows that pHH-GM1 induced significantly enhanced His-HA-ALPK1 gene expression in a western blot analysis of transfected HEK293T cells compared with six commercial plasmids (pCDNA3.1, pEGFP-C2, pFN21K, pME-HA, pFLAG-CMV-2 and pCMV6-entry) and this experiment was repeated three times independently with similar results (supplemental Fig. 6). Figure 4b shows the fluorescence intensity of turbo GFP (tGFP) after transfecting cells with pHH-GM1 encoding tGFP (ptGFP-HH) compared with that of a commercial vector (pEGFP-C2). The results revealed that whereas ptGFP-HH transiently transduced HEK293T cells, as evidenced by the appearance of fluorescence indicating expression of the tGFP gene, there was no tGFP expression in the mock transfection cells (pHH-GM1 vector only). In a further comparison of GFP-ALPK1 expression levels in HEK293T cells transfected with the CMV promoter-driven vectors (pEGFP-C2 and ptGFP-HH), the ALPK1 gene expression induced by ptGFP-HH was observed to be better (Fig. 4c; box 3 and 4) than that induced by pEGFP-C2.

### Scaling up for protein production in HEK293-F and CHO-S cells

**Figure 5:**
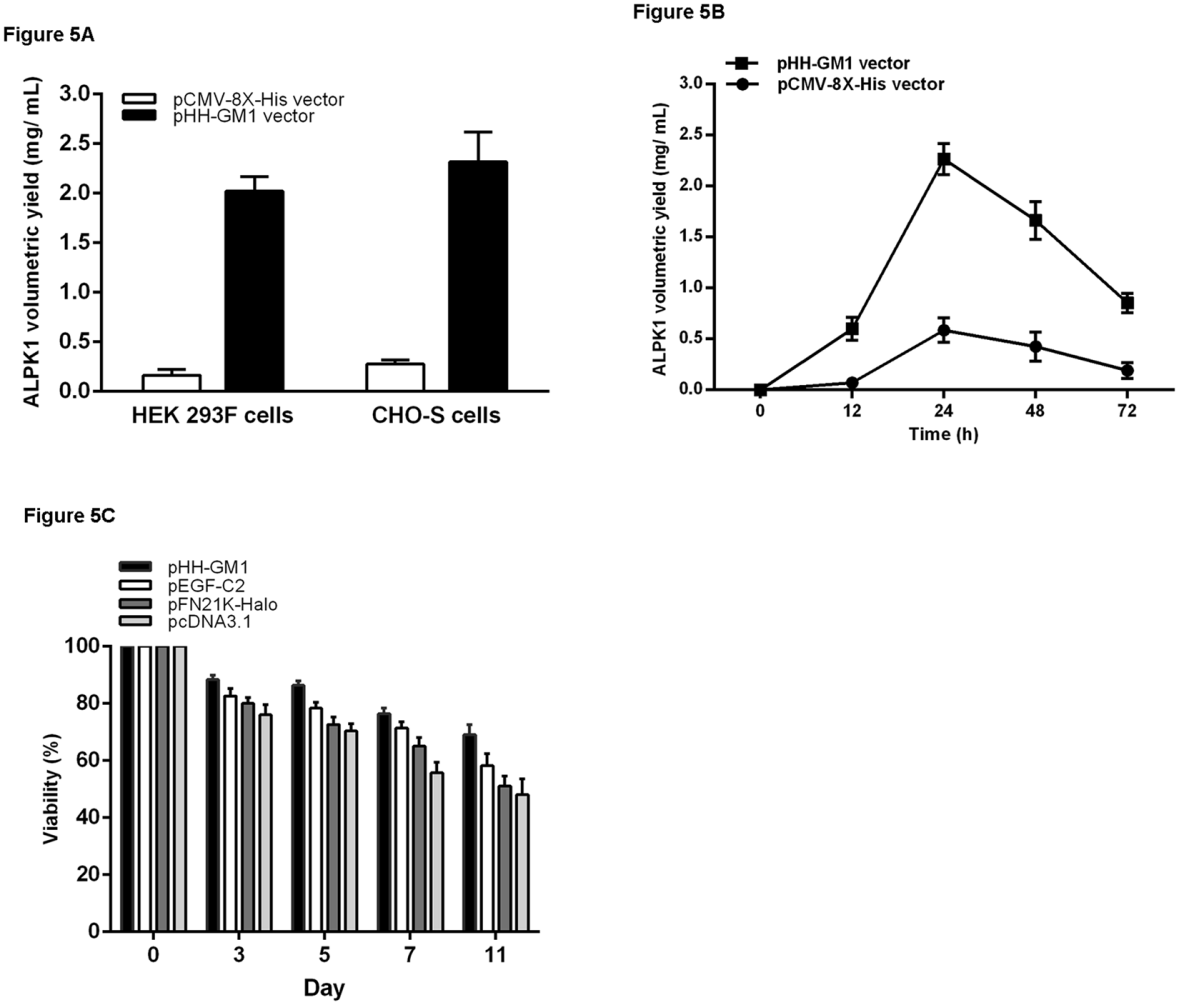
(**A**) The human ALPK1 protein expression level was measured 48 h after the start of transfection. Transfection of FreeStyle 293-F cells or FreeStyle CHO-S cells in 1 L Erlenmeyer cell culture flasks. The cells at a density of  6 × 10^7^ cells/mL in 500 mL FreeStyle 293-F expression medium and FreeStyle CHO expression medium, respectively, were transfected with pHH-GM1-ALPK1 or pCMV-8 X-His at a DNA:FreeStyle MAX Reagent ratio of 1:2.5 with a final DNA concentration of 5 μg/mL in the culture medium. At 6 h post-transfection, the culture was diluted with one volume of culture medium. (**B**) The ALPK1 protein production at different time points. (**C**) The cell growth rate: 1 × 10^6^ cells/mL FreeStyle 293-F cells were transfected with 2 μg/mL of plasmid DNA (pHH-GM1, pEGFP-C2, pFN21K-Halo and pcDNA3.1) and examined at various times (3, 5, 7 and 11 days).

To optimize the pHH-GM1 vector for transient expression of ALPK1 in large-scale experiments, we examined its effect on yield time and cytotoxicity in two mammalian cell lines (FreeStyle 293-F and CHO-S). We tested pHH-GM1 and pCMV-8X-His for their suitability as DNA delivery vehicles for transfection of HEK293-F and CHO-S cells adapted to serum-free suspension growth. The cells were distributed into 1 L Erlenmeyer flasks at a density of 6 × 10^7^ cells/mL in FreeStyle CHO expression medium and transfected with pCMV-8X-His or pHH-GM1 using DNA:FreeStyle MAX Reagent (w:w) using ratios ranging from 1:1 to 1:4. The transient production of pHH-GM1-ALPK1 was tested by standard supplementation with one volume of medium, and volumetric yields of 2 mg/L and 2.3 mg/L were achieved in HEK293-F and CHO-S cells, respectively. The amount of r-protein obtained after transfection with pHH-GM1 was markedly higher than that obtained after transfection with the corresponding pCMV test vector (pCMV-8X-His) (Fig. 5a). We try to prove the ptGFP-HH vector of stable and scalable for transient protein expression in different volume of culture medium (0.1, 0.25. 0.5 and 1 L). In different volume of culture medium, the result shown that amount of GFP expression induced by ptGFP-HH was observed to be higher yields and stable (Supplemental Fig. 4) than that induced by pEGFP-C2. After transfection with pEGF-C2 vector, culture in over 0.5 L medium was observed with decreasing GFP level. When we tested for the optimal expression timeframe, it was observed for both vectors (pHH-GM1 and pCMV-8X-His) that the highest yields were obtained at 24 h, and the highest overall yield was obtained within 72 h after transfection. In total, pHH-GM1-ALPK1 achieved significantly (*P* = 3.23 × 10^−8^) higher yields than pCMV-8X-His-ALPK1 after adjusting for time. At 24 h, pHH-GM1 showed the highest yield at approximately 2.3 mg/L, which was more than 2.5-fold that obtained with pCMV-8X-His. (Fig. 5b).

### Cellular viability assessment using four different vectors

In terms of vector toxicity against mammalian cell viability post-transfection, we tested four vectors (pHH-GM1, pEGFP-C2, pFN21K-halo, pcDNA3.1) by applying Tukey’s post hoc test and found a significant (*P* = 5.96 × 10^−15^) difference among groups after adjusting for day, showing that cell viability after transfection with pHH-GM1 was significantly higher (*P* < 0.0001) than that after transfection with the other vectors across pairwise comparisons. By day 11, the average viability was approximately 70% after transfection with pHH-GM1 vector compared to 60% after transfection with pEGFP-C2, pFN21K-halo and pcDNA3 vectors (Fig. 5c).

## Discussion

We demonstrated that the novel pHH-GM1 vector consisting of the ctEF-1 first intron in combination with two enhancers (CMV followed by SV40) could be used to express six different genes as well as for co-transfection of cells with multiple plasmids. The ALPK1 gene was selected for detailed study, and overall, we found that pHH-GM1 vector was superior to six commercial vectors in terms of increased protein expression and GFP fluorescence, higher yields with two cell types (HEK293-F, CHO-S), higher yields across the expression timeframe, and better cell viability after transfection. Thus, the multiple capabilities of the pHH-GM1 vector might enable more complete protein characterization, which in turn should broaden our understanding of protein cellular functions. In our previous studies, we solved many of the experimental limitations of exploring cellular biological functions using this vector. We utilized the pHH-GM1 vector to produce a full length protein, ALPK1, that is difficult to express according to our past experiences. We chose this new developmental expression system try to turn the limit which, difficult to express proteins in a mammalian cell for past. In our data shown, this new system had observed still exists some disparity in protein levels though it maybe could be used in the widely mammalian system. We assumed that different genes used in pHH-GM1 system attributed to this phenomenon. Therefore, maybe there is no single expression system and cell line that is always the best performer until now. However, we successful obtained the ALPK1 r-protein by pHH-GM1 and used it to complete for various molecular functional assays, demonstrating that ALPK1 phosphorylates a motor protein, which also mediates a pro-inflammatory cytokine sorting process via exocytic transport to the cellular membrane^13^. Based on a report that heterologous introns significantly improve gene expression^14^, we chose the first intron of human EF-1α for vector modification. The EF-1 protein is one of the most abundant proteins in most tissues, and the EF-1 promoter drives high-level gene expression in various cell types^9^. Moreover, we inserted a GFP gene under the CMV promoter with or without the wtEF-1α first intron in combination with double enhancers and compared the GFP expression level driven by the two constructs. Although GFP expression appeared to increase slightly with inclusion of the wtEF-1α first intron, there was no significant difference between the downstream CMV promoter activity with or without the wtEF-1α first intron. The results demonstrated that GFP reached a maximum protein expression level in HEK293T cells, perhaps only as a result of the double enhancers. We also truncated the size of the EF-1α first intron from 592 (wt) to 367 bp (ct partial), and we retained the major five Sp1 and a single Apl binding regions in the first intron of the EF-lα gene. A comparison of the wt and the ctEF-1α, both with double enhancers, showed that the protein levels correlated well with the transcript levels if a gene-specific and cell-independent RNA-to-protein conversion factor is introduced. The results showed that the ctEF-1α induced significantly higher ALPK1 gene and protein levels than the wtEF-1α intron. We surmise that the increased efficiency of the transgene transcriptional level was due to reducing the EF-1α intron size from 592 to 367 bp. We also tried to truncate the N-terminal portion of the EF-lα intron (ntEF-lα first intron, +1 to +350) and inserted it downstream of the CMV promoter, and the results showed that the ALPK1 expression signal was very weak compared with that induced by the wtEF-1α intron vector (data not shown). The ntEF-lα first intron lacking the Spl sites was compared with the wtEF-lα intron, and the results indicated that none of the putative Sp1 binding sites are necessary for CMV promoter activity^10^. Interestingly, when we combined the two ctEF-1α introns and the double enhancers, the ALPK1 protein level was not increased but instead reduced compared with that induced by the wtEF-lα first intron and one ctEF-1 intron. A major difference was found between the two ctEF-1α first intron vectors, the ctEF-1α first intron vector and the wtEF-1α first intron vector (commercial systems). However, it was unclear why further multiples of the ctEF-1 first intron beyond two, which should increase the transcription factor binding sites, had reduced expression efficiency. Similarly, in our preliminary experiment, we found that the use of triple enhancers was inferior to the use of double enhancers. Therefore, we chose the optimum elements of the ctEF-1 first intron and double enhancers for this experiment because this type of construct had high protein expression efficacy in all our tests. It is possible there is complex regulation and multiple promoter:enhancer interactions that we have not considered. While we showed that the pHH-GM1 vector can be used for six genes, the expression may vary for other genes. In addition, the transfection was transient because Lipofectamine was used as opposed to a longer-term viral vector-mediated transduction. The data presented herein demonstrate that a human CMV vector that includes the ctEF-1α and double enhancers achieved the highest protein expression levels in HEK293-F/CHO-S cells in both transient and stable transfections, even for large-scale systems. To test promoter interference and compatibility, we performed co-transfection of dual vectors using our developed vector system. However, notably, the robust and promiscuous activity of the CMV promoter could cause promoter interference^15^ or transcriptional silencing^16^. The CMV enhancer/promoter is notorious for “cross-talk” with other promoters because it contains many transcription factor binding sites (SP1, AP2, NF-κB, and CREB binding sites)^17^. The effects of the representative genes on mRNA expression and protein levels have been observed with numerous different genes of interest (data not presented), but for some genes, they are insufficient, and the capacity of our developed vector to express specific genes cannot be guaranteed. With our vector, one genetic construct can be used in various *in vivo* and *in vitro* assays, including cellular imaging for localization and trafficking studies and analysis of protein interactions. This new vector could solve some of proteins expression problem, especially difficult expression proteins in mammalian cells e.g. kinase and high molecular weight protein. Although the pGM1-HH vector cannot necessarily suit for each gene expression, this vector incorporates all of the advantages of pCMV vectors for expression, while also increasing ease-of-use for target gene cloning and manipulation of plasmid DNA. We also consider the kind of experiments requires, adding two tags and a proteolytic fusion tag recognition site of this vector in order to further understand the function of a specific gene and good for protein purification (Supplementary Fig. 7). Moreover, we sent our purified ALPK1 protein to a commercial customized antibody service (AllBio Science, Inc), who induced the polycolonal antibody in mice and have verified that these antibodies are ALPK1-specific (Supplementary Fig. 8). Thus, the capabilities of the pHH-GM1 vector improve gene and protein expression levels, and thus, this vector is applicable to protein drug discovery, antibody production and vaccine development. The present modification in the transposition-based vector through ctEF-1 first intron and double enhancer integration is cost effective, saves considerable time, and addresses the issue of protein expression in mammalian systems.

## Methods

### Vector design and construction

pEGFP-C2 and pFN21K-Halo basic vectors were purchased from Promega (Madison, WI) and Clontech (Palo Alto, CA), respectively. pcDNA3.1 was purchased from Invitrogen (Carlsbad, CA), pME-HA purchased from Lucigen (Middleton, WI), pFLAG-CMV-2 purchased from Sigma-Aldrich (St. Louis, MO), pCMV6 entry purchased from OriGene (Rockville, MD) and pCMV-8X-His was obtained from Dr. Ma’s Lab (GRC, Academia Sinica, Taiwan). Supplemental Fig. 1 shows the designed pHH-GM1 plasmid vector backbone based on pFN21K-Halo, from which we retained the CMV intermediate early promoter, the SV40 early mRNA polyadenylation signal, the pUC origin of replication, and the kanamycin resistance gene. From downstream of the CMV intermediate early promoter, the chimeric intron through the stop codon was excised using *Hind*III and *Nhe*I, and a new synthetic DNA fragment consisting of the ctEF-1α (GenBank no: LT727183.1; 367 bp, Supplemental Fig. 2), the Kozak consensus translation initiation site, a flexible linker, the TEV cleavage site, 6X-histidine, the human influenza HA epitope, and a multiple cloning site was inserted, flanked by *Hind*III and *Nhe*I sites. The region downstream of the stop codon through the SV40 late polyadenylation signal was excised using *Pme*I and *Bam*HI, and a new synthetic DNA fragment consisting of the SV40 late polyadenylation signal, CMV enhancer (479 bp: accession no. AJ318513 (159–637)) and SV40 enhancer (319 bp: accession no. AY864928 (2156–2474)) was inserted, flanked by *Pme*I and *Bam*HI sites.

The ptGFP-HH plasmid vector was generated following digestion of the linker region between *Nhe*I and *Pme*I from the pHH-GM1 vector, and a synthetic fragment consisting of the Kozak consensus translation initiation site, a flexible linker, the TEV cleavage site, 6X-histidine, the human influenza HA epitope, tGFP, and the multiple cloning site was inserted, flanked by *Nhe*I and *Pme*I sites. The various genes (e.g., full-length ALPK1)(Supplementary Fig. 5) were subcloned into the multiple cloning site position, flanked by the *Aisi*I and *Pme*I sites of the pHH-GM1 or ptGFP-HH vector. All constructs were validated by sequencing.

### Expression of ALPK1 in transiently transfected HEK293T cells

The adherent human embryonic kidney (HEK) cell line HEK293T was obtained from Bioresource Collection and Research Center (BCRC, Taiwan) and cultured in α-MEM containing 100 U/mL penicillin/streptomycin and 10% fetal bovine serum at 37 °C, 5% CO_2_ and 95% humidity. The HEK293T cells were transfected using Lipofectamine 2000 (Invitrogen) as described previously^18^. Briefly, the ALPK1 full-length fragment was inserted into a plasmid vector (pHH-GM1, pEGFP-C2, pFN21K-Halo or pcDNA3.1), and Lipofectamine 2000 (Invitrogen) was used for transient transfection. The ratio of DNA to Lipofectamine 2000 was optimized for cell viability and transfection efficiency. The following ratio was used: 2.5 μg of DNA was combined with 10 μl of diluted Lipofectamine 2000 per 10 cm^2^ of 80–90% confluent HEK293T cells. The plasmid DNA and Lipofectamine 2000 were separately diluted in opti-MEM and incubated for 5 min. They were combined along with serum-free α-MEM and incubated with cells for 5 h and then replaced with complete α-MEM. The cells were cultured for 24 h prior to harvest.

#### Preparation of protein for large-scale production

For large-scale expression, suspension-adapted FreeStyle 293-F cells and FreeStyle CHO-S Cells (Invitrogen, Carlsbad, CA) were routinely cultured in Gibco FreeStyle 293-F expression and FreeStyle CHO expression medium in 1 L disposable Erlenmeyer tissue culture flasks with vented caps (Corning Inc., Corning, NY), respectively, at 37 °C in a 5% CO_2_ atmosphere at 120 rounds per minute (rpm) on an orbital shaker platform (MIR-S100C shakers with universal platforms, SNAYO Electric).

### Cell viability analysis using flow cytometry and propidium iodide staining

HEK293T cells were transfected using four different vectors and collected at different time points. The cells were transfected using the Lipofectamine 2000/plasmid DNA method described above. Staining with propidium iodide (PI) (Thermo Fisher Scientific) and flow cytometry staining buffer (R&D Systems) was performed followed by flow cytometry as described previously^19^. In brief, the cultured HEK293T cells were harvested, washed with cold PBS and resuspended in 40 μL 1X Annexin V binding buffer, followed by PI staining for another 15 min at 4 °C in the dark. The stained cells were analysed via flow cytometry within 30 min.

### RNA isolation and cDNA synthesis

Total RNA was extracted from HEK293T cells in both the treatment and control groups with TRIzol (Invitrogen, Carlsbad, CA) according to the manufacturer’s instructions. RNA concentrations were calculated based on OD readings, and the 260/280 values of all RNA samples were ≥1.8 according to measurements taken with a NanoDrop 2000 spectrophotometer (Thermo). Total RNA was converted to cDNA using a High Capacity cDNA Reverse Transcription Kit with random primers (Applied Biosystems, Warrington) according to the manufacturer’s instructions.

### Real-time PCR

Real-time PCR analysis was applied to elucidate changes in tGFP mRNA expression using *SYBR* Green (Applied Biosystems, Foster, CA), primers tGFP forward (5′GGCACCCTGAACGGCGTG 3′), reverse (5′ GGTGCCGAAGTGGTAGAA 3′), annealing temperature (Tm) of 60 °C and an ABI StepOne™ Real-Time PCR System (Applied Biosystems, Foster, CA). The relative quantification was normalized against ACTB expression. All procedures were performed according to the recommendations of the manufacturers, and the quantitative data shown are presented as the means of triplicate experiments.

### Western blotting

24 hr after transfection, cell extract was harvested using either RIPA lysis buffer containing 25 mM Tris-HCl (pH 7.2), 125 mM NaCl, 1% NP-40, 0.5% sodium deoxycholate, 1 mM EDTA and 5% protease inhibitor cocktail (Roche) or SDS-sample loading buffer. The protein concentration in each sample was estimated with a Bio-Rad protein assay (Hercules, CA). An equivalent amount of protein from each condition of the cell extracts was loaded onto a reducing 8% SDS-PAGE gel and transferred to a polyvinylidene difluoride (PVDF) membrane (Millipore, Bedford, MA). After the membrane was blocked with TBS/5% bovine serum albumin (BSA), antibodies against ALPK1 (1:500, Gentex), GFP (Gentex), HA (1:1000, Cell Signaling Technology), FLAG (1:1000, Sigma) β-actin (1:1000, Gentex) and GAPDH (1:5000, Merck Millipore) were incubated with the membranes at room temperature for 1 h. The resulting immunocomplexes were detected using HRP-conjugated secondary antibodies and developed with an enhanced chemiluminescence reagent (Amersham Biosciences).

## Electronic supplementary material

Supplementary Information
