# Supplementary material for: Regulatory elements in vectors containing the ctEF-1α first intron and double enhancers for an efficient recombinant protein expression system
Source: Sci Rep. 2018 Oct 18;8:15396. doi: 10.1038/s41598-018-33500-0 (PMC6193983; doi:10.1038/s41598-018-33500-0)
Supplement: Supplementary file 1 — Supplementary Information [file 41598_2018_33500_MOESM1_ESM.pdf]

Regulatory elements in vectors containing the ctEF-1α first intron and double enhancers for an efficient recombinant protein expression system

Chi-Pin Lee,[1¶] Albert Min-Shan Ko,[2¶] Shang-Lun Chiang,[1,3] Chi-Yu Lu,[4] Eing-Mei Tsai,[5] Ying-Chin Ko[1*]

[1]Environment-Omics-Diseases Research Center, China Medical University Hospital, China Medical University, Taichung, 40402 Taiwan

[2]Key Laboratory of Vertebrate Evolution and Human Origins of Chinese Academy of Sciences, IVPP, CAS, Beijing, 100044 China

[3]Department of Health Risk Management, College of Public Health, China Medical University, Taichung, 40402 Taiwan

[4]Department of Biochemistry, College of Medicine, Kaohsiung Medical University, Kaohsiung, 80708 Taiwan

[5]Graduate Institute of Medicine, College of Medicine, Kaohsiung Medical University, Kaohsiung, 80708 Taiwan

¶Chi-Pin Lee and Albert Min-Shan Ko contributed equally to this study are considered co-first authors.

*Correspondence to: Ying-Chin Ko, MD, PhD, Professor, Environment-Omics-Disease Research Centre, China Medical University Hospital, China Medical University, Taichung, 40402 Taiwan. 2 Yude Road, Taichung, 40447 Taiwan. TEL 886-4-2205-2121 ext.7322.

E-mail: ycko0406@gmail.com

Supplemental

Figure 1. Schematic structures of constitutive expression vectors with pHH-GM1 derived from pFN21K plasmid vector.

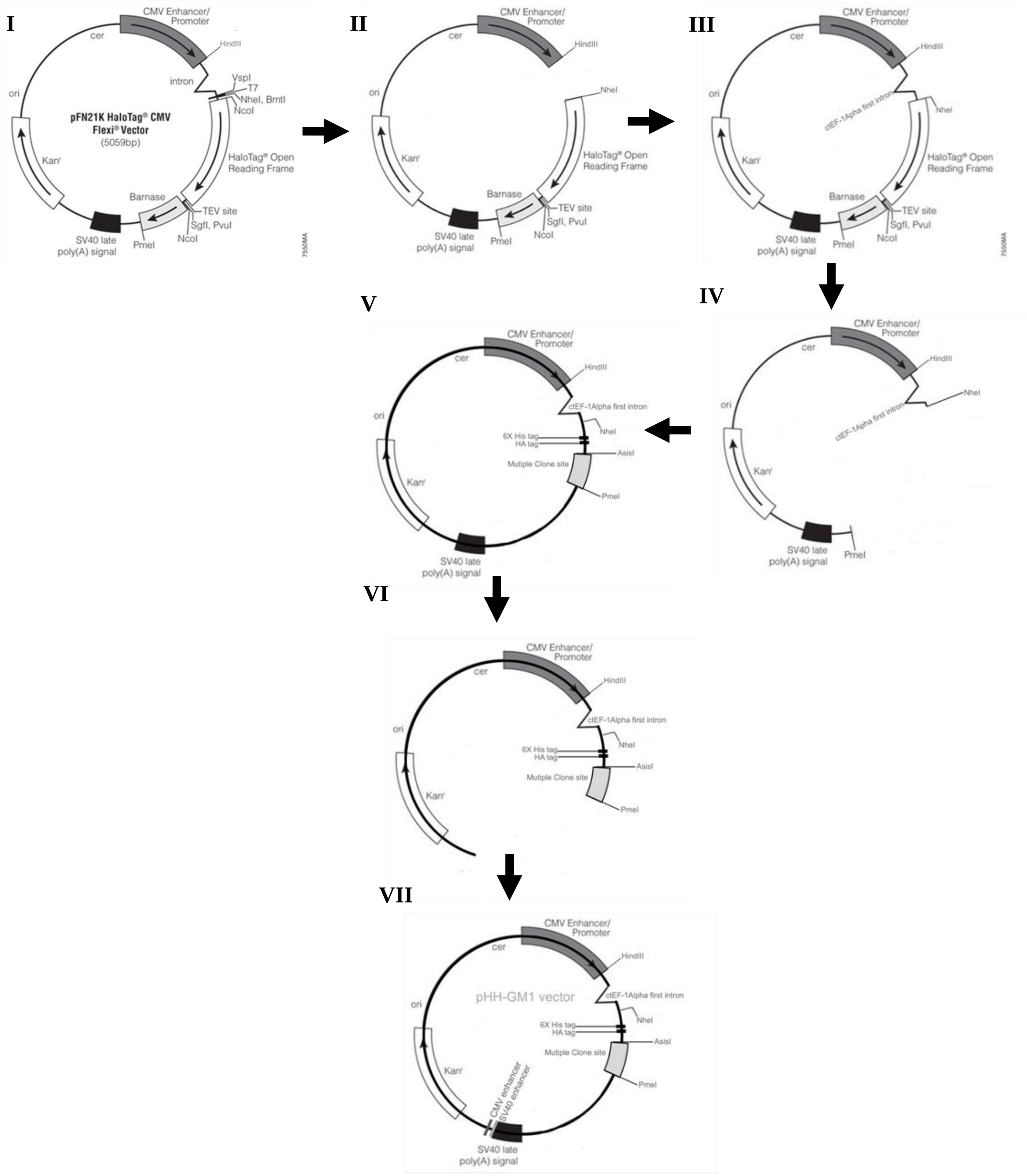

***Figure 2. The sequence of C-terminal elongation factor-1α first intron Gene (367* bp)**

CGGTTTTTGGGGCCGCGGGCGGCGACGGGGCCCGTGCGTCCCAG
CGCACATGTTCGGCGAGGCGGGGCCTGCGAGCGCGGCCACCGAG
AATCGGACGGGGGTAGTCTCAAGCTGGCCGGCCTGCTCTGGTGC
CTGGCCTCGCGCCGCCGTGTATCGCCCCGCCCTGGGCGGCAAGG
CTGGCCCGGTCGGCACCAGTTGCGTGAGCGGAAAGATGGCCGCT
TCCCGGCCCTGCTGCAGGGAGCTCAAAATGGAGGACGCGGCGCT
CGGGAGAGCGGGCGGGTGAGTCACCCACACAAAGGAAAAGGGCC
TTTCCGTCCTCAGCCGTCGCTTCATGTGACTCCACGGAGTACCG
GGCGCCGTCCAGGCA

Putative Spl binding sites are red, (G/T)(G/A)GGC(GPT)(G/A)

Apl binding sites are blue

Figure 3. Design and mapping of pHH-GM1 vector with ALPK1 full length gene.

The pHH-GM1-ALPK1 was validated by forward (A) and reverse (B) sequences

(A)

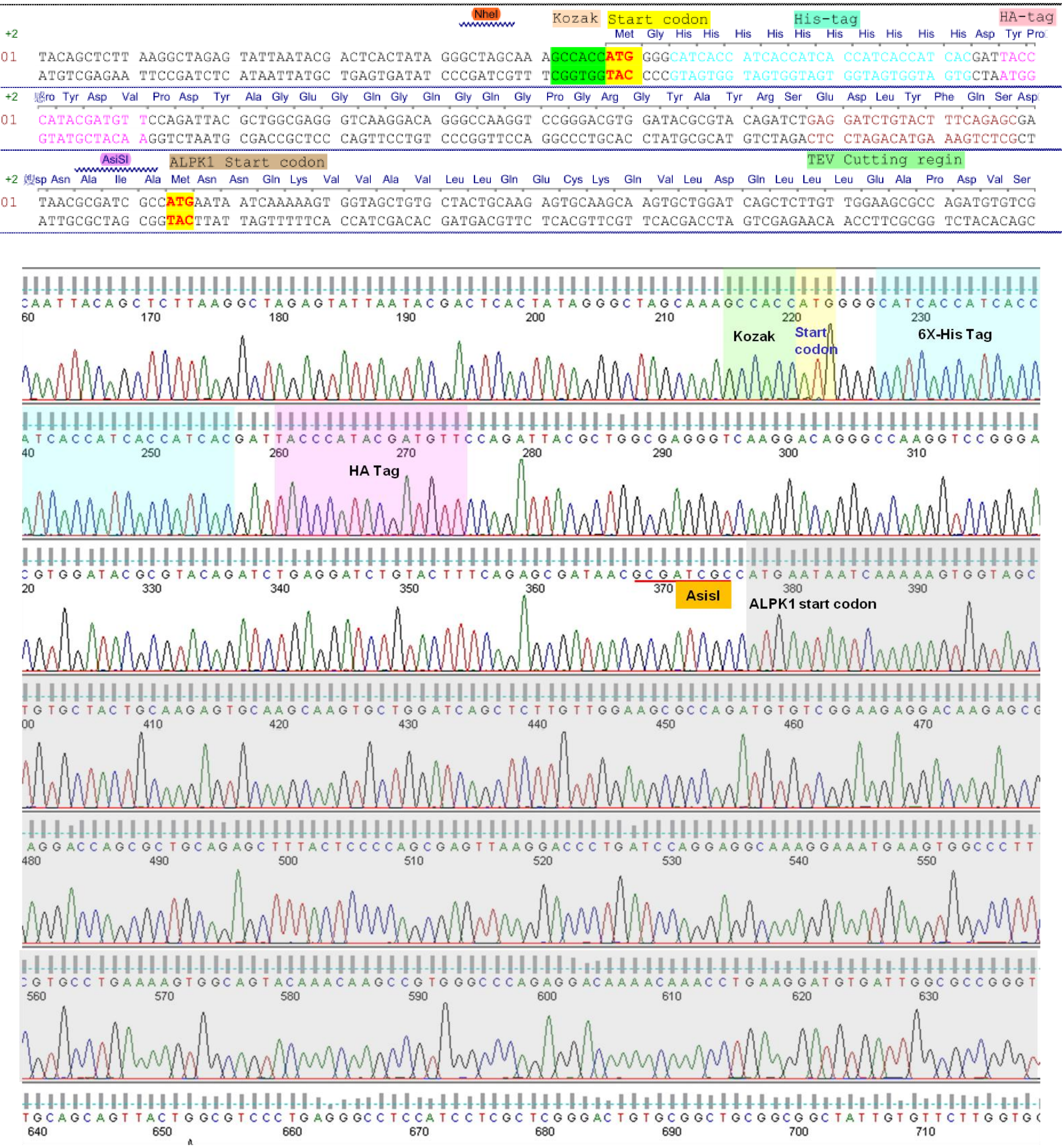

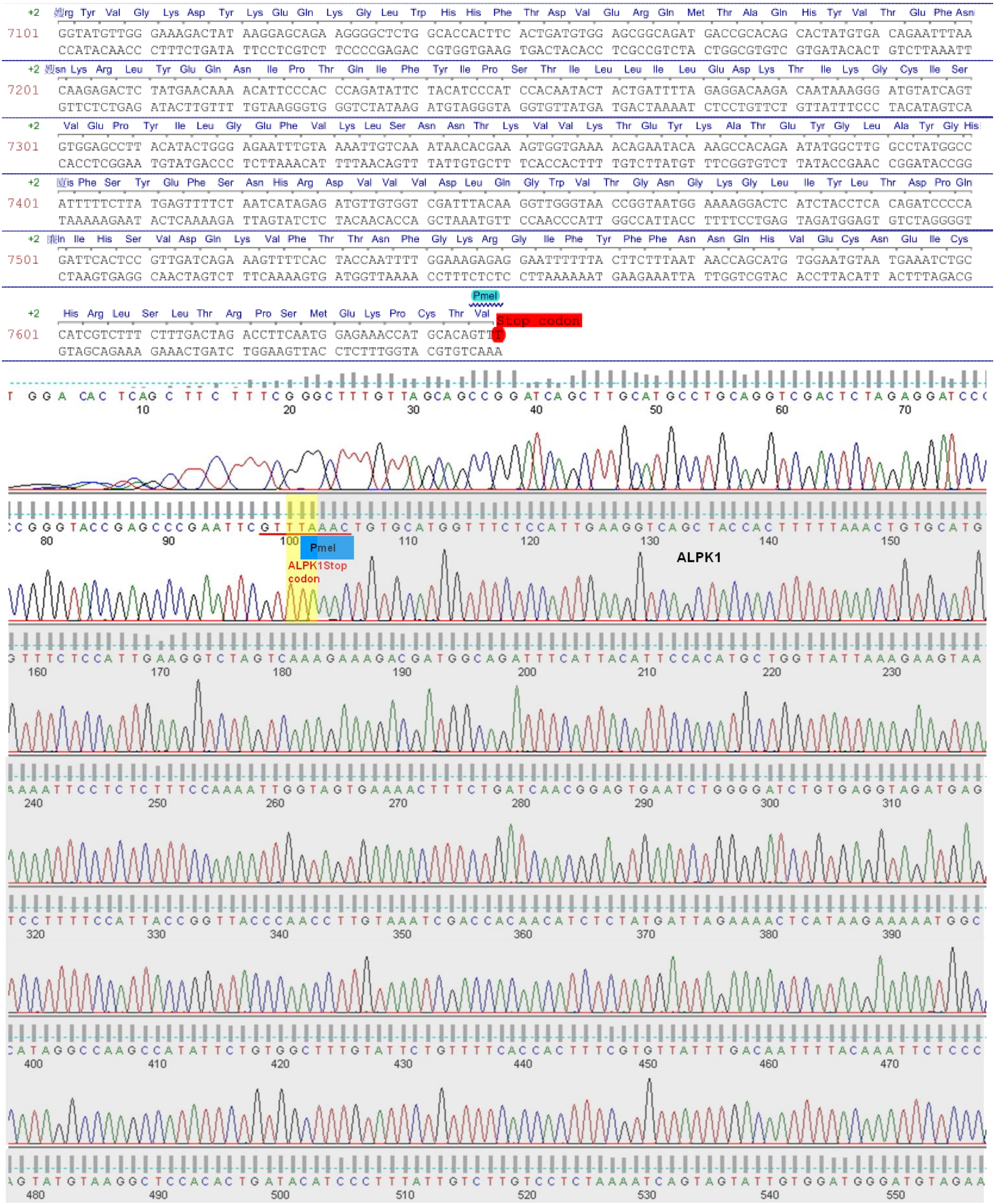
PmeI
Stop codon
PmeI
ALPK1Stop codon
ALPK1

Figure 4. Comparison of GFP expression stability of two vectors into different volume of culture medium

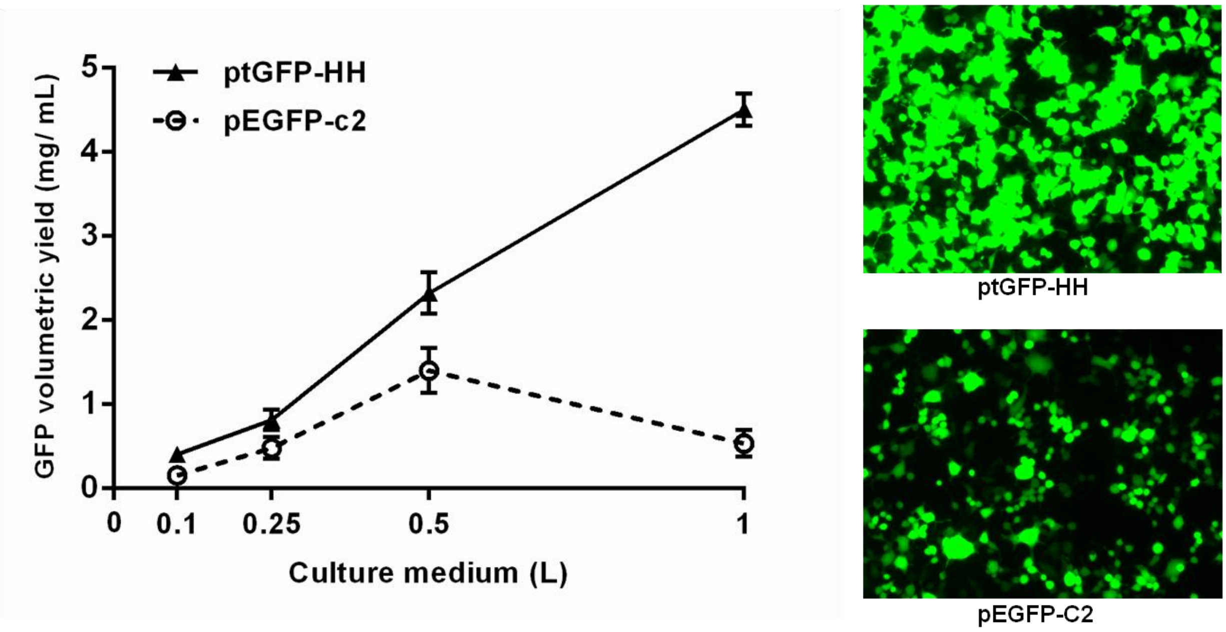

HEK293F cells transiently transfected with plasmid ptGFP-HH or pEGFP-C2 for GFP expression in different volume of culture medium (0.1, 0.25. 0.5 and 1L). Both DNAs harbour an identical gene expression cassette encoding GFP and were transfected using equivalent plasmids. Images were taken at 24 h post-transfection. Magnification, 10x objective. GFP expression detected under a fluorescence microscope.

Figure 5. The ALPK gene full length sequenced

ALPLK_01-813F

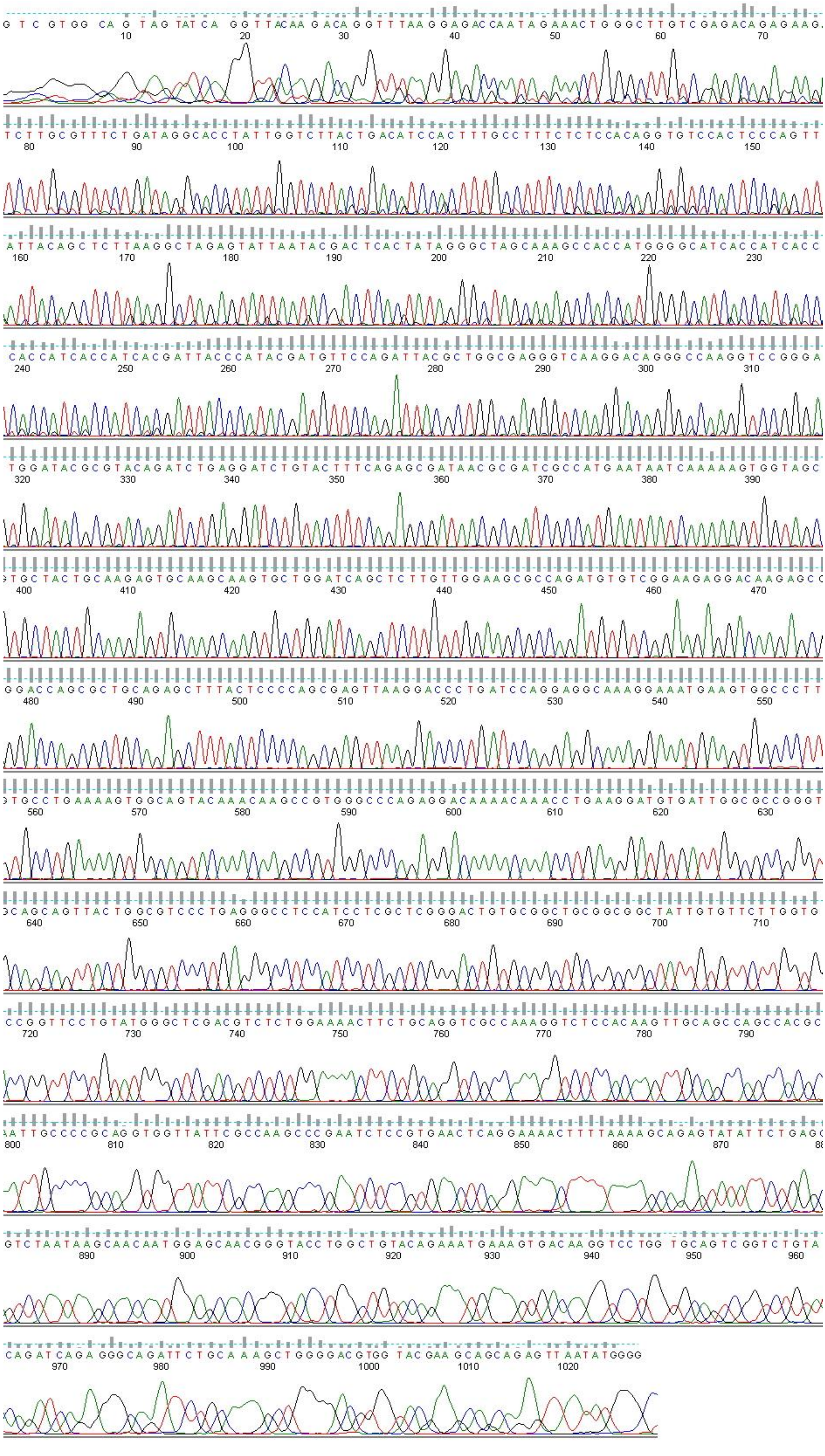

ALPLK_747-1707F

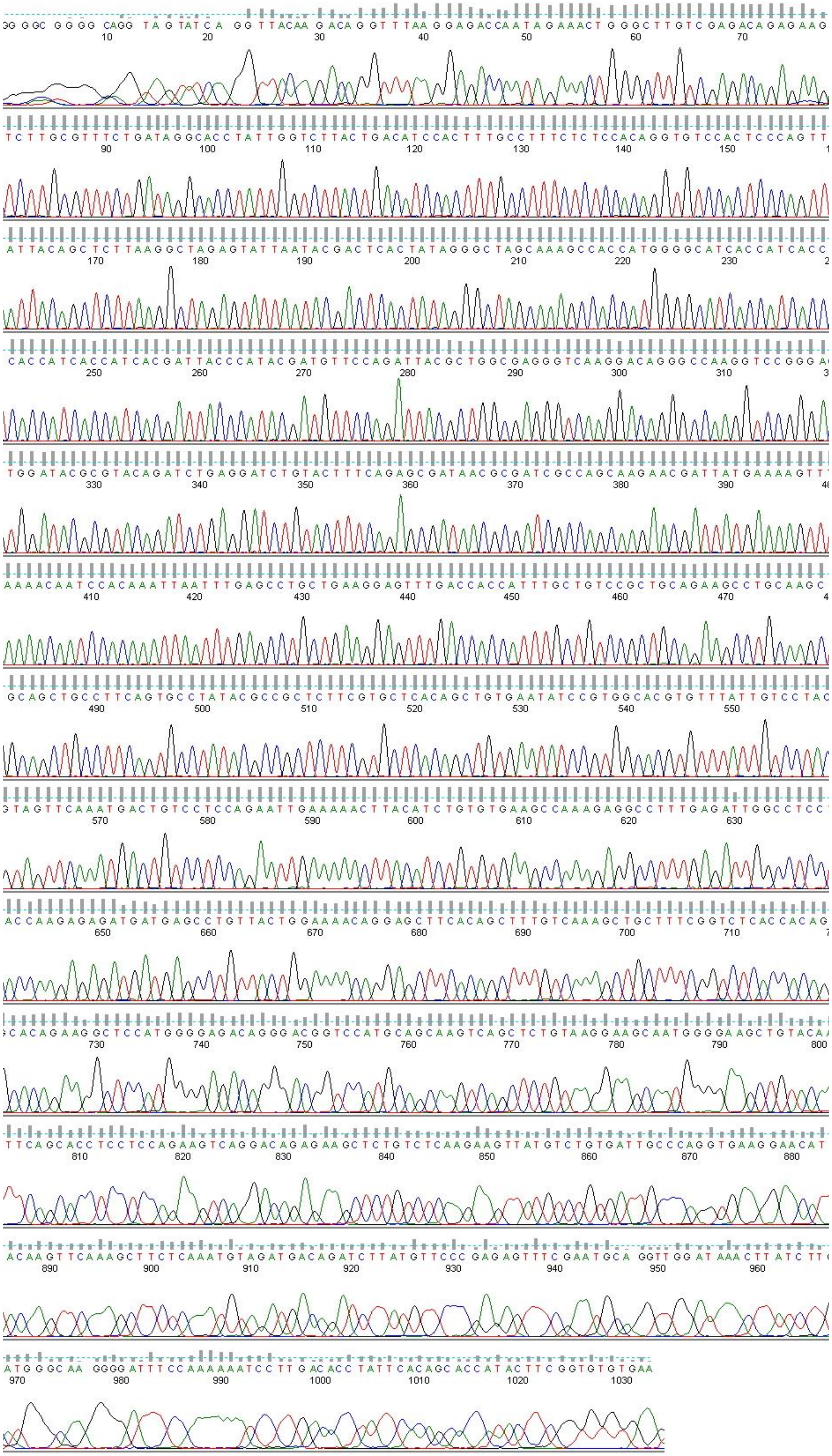

ALPK1 full length_1982-2977F

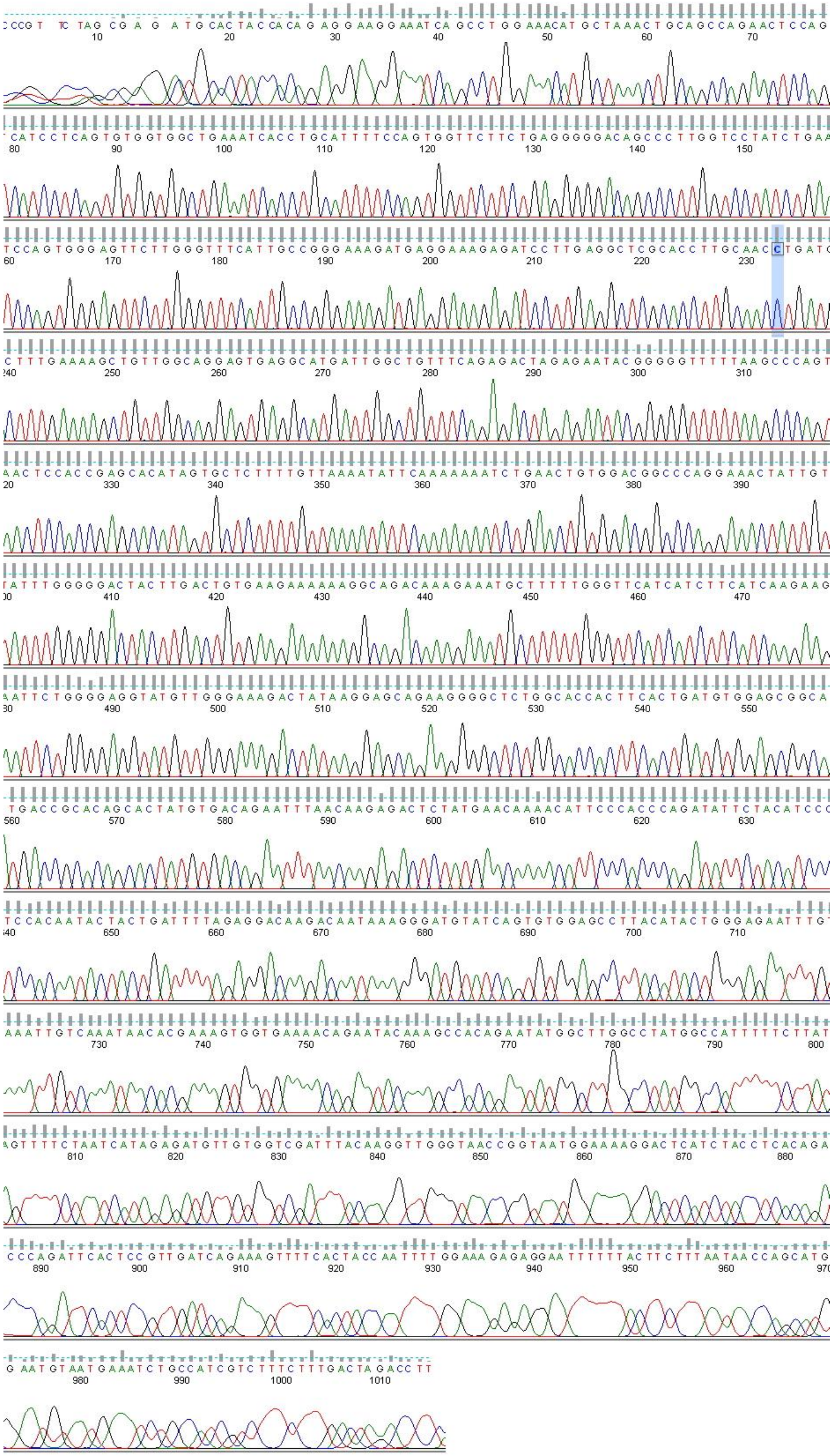

ALPK1 full length_2848-3732F

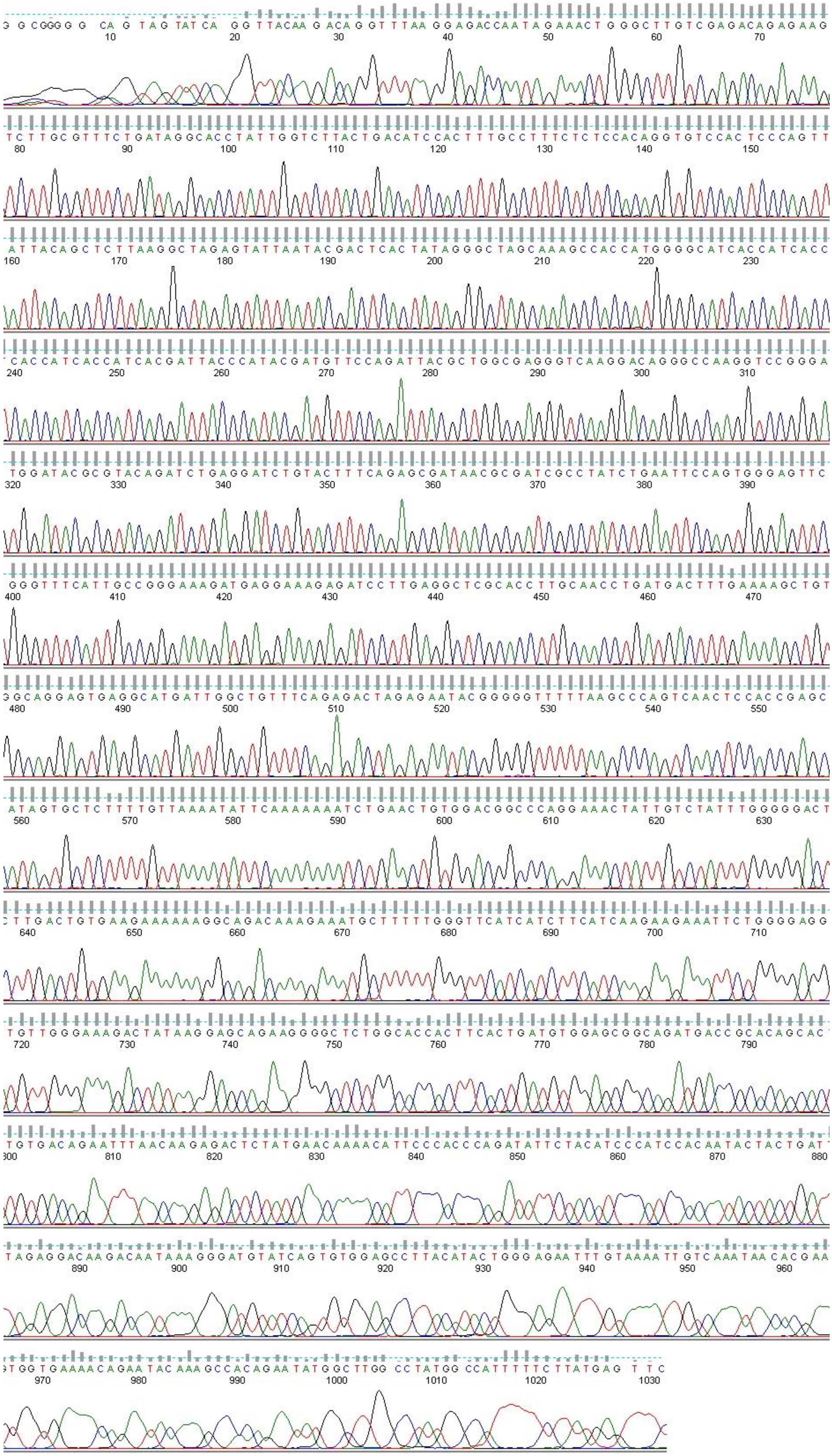

Figure 6. Comparison of the protein expression of ALPK between pHH-GM1 and various commercial vectors were transfected in mammalian cells.

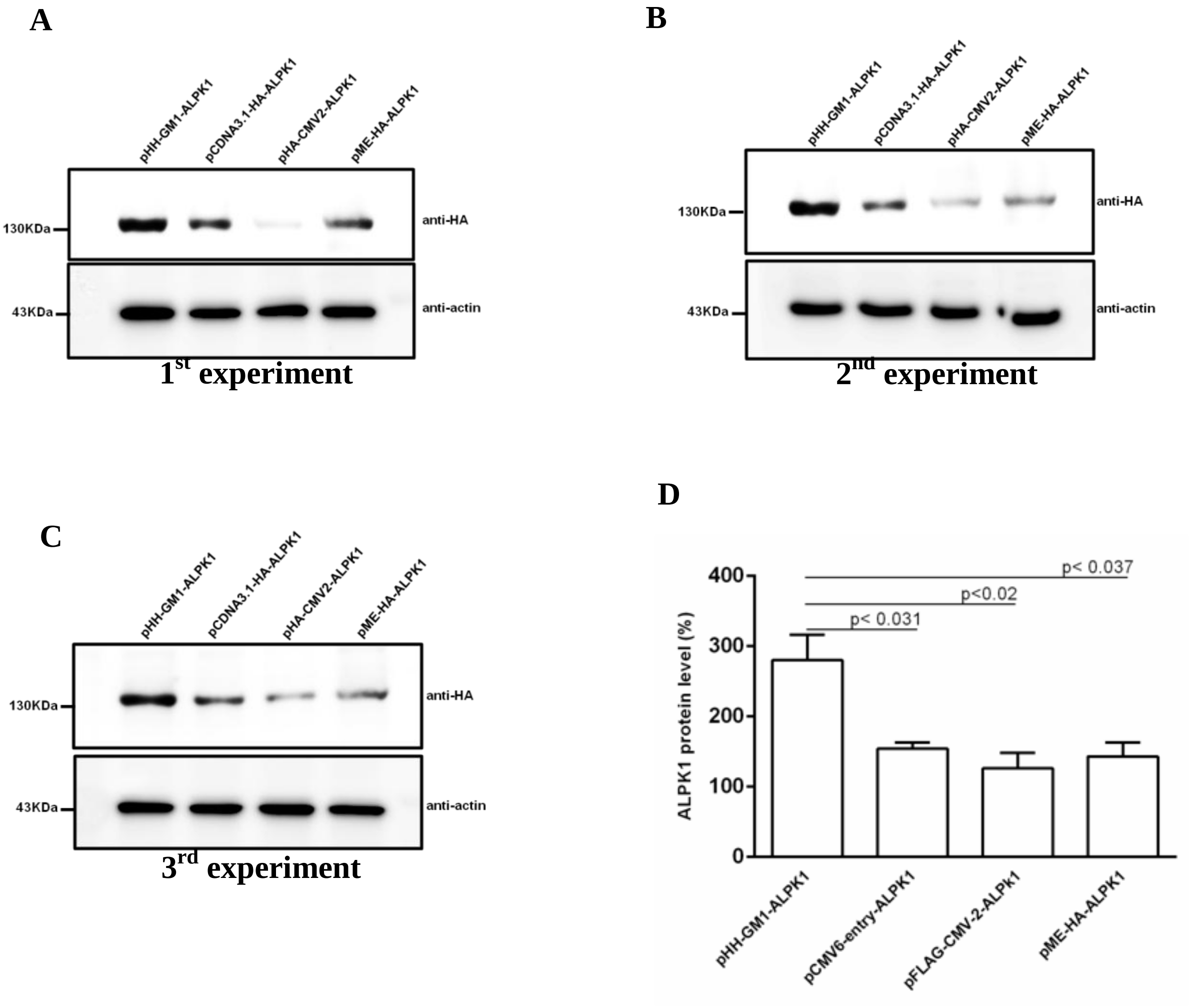

This figure shows (A), (B) and (C) the pHH-GM1 and different commercial vectors (pcDNA3.1, pFLAG-CMV2, pME-HA) were used for (D) comparing ALPK1 protein levels in HEK293T cells. The data presented represent the combined results of three independent experiments. 30 µg of total protein extract was loaded for each lane. One-way analysis of variance and Wilcoxon signed-rank test were conducted to examine in relation between vectors and different protein levels. These data are expressed as the mean ± SD (n=3). *P<0.05

Figure 7. Purification of ALPK1 full length protein using affinity chromatography

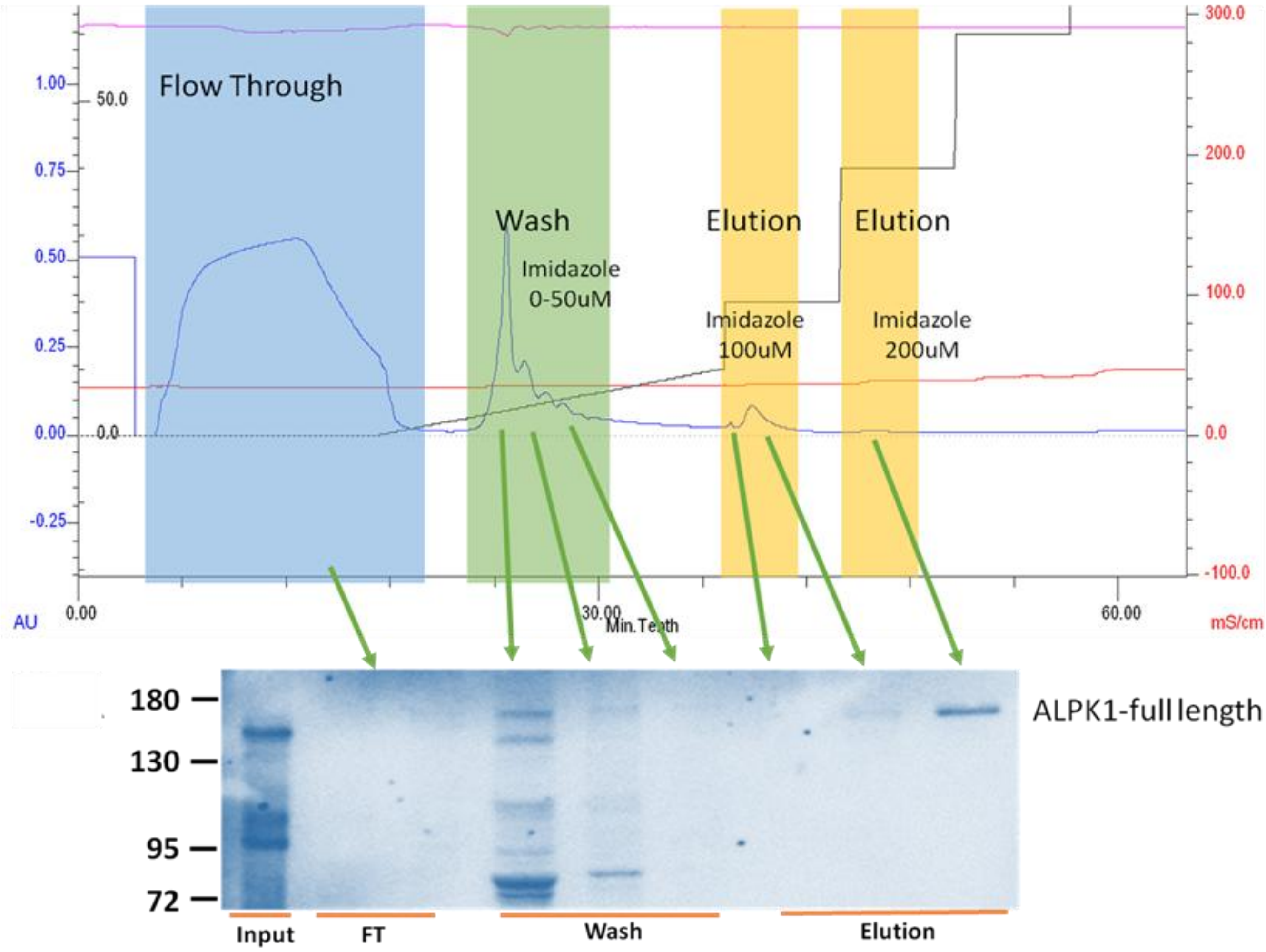

The fusion protein His-HA-ALPK1 expressed by plasmid pHH-GM1 vector for 24 h and affinity purified using Histidine-Sepharose columns (Bio-Scale Mini Profinity IMAC Cartridges, Du bendorf) by liquid chromatography (BioLogic DuoFlow; BioRad, Reinach). The frozen cell pellets were resuspended in Buffer A with 1 mM phenyl methyl sulfonyl fluoride (PMSF), and 1X EDTA-free protease inhibitor cocktail tablets (Roche, Basel), 1µg/ml Dnase I on ice for 20 min. Cells were sonicated for 5 min on ice, and the suspension was centrifuged at 4℃ (13,500 rpm for 30 min). The supernatant from this centrifugation was loaded onto a Ni-NTA column

equilibrated in buffer A by manually injection into a sample loop. The column was washed with 10 column volume buffer A containing 30 mM imidazole and then the protein was eluted with 4 column volume buffer containing 200 mM imidazole. The collected fractions were subjected to SDS-PAGE and stained with Coomassie Blue.

Customized Antibody Service Data Sheet

Cat No: ABCSMM151116H02011LINDA001 [PHc-2-1B]
ABCSMM151116H02011LINDA001 [PHc-3-10C]
ABCSMM151116H02011LINDA001 [PHc-3-12A]

**Product Name:**	Anti-ALPK1 Polyclonal Antibody
**Host:**	Mouse
**Product type:**	Polyclonal Antibodies
**Colnality:**	Polyclonal
**Immunogen:**	ALPK1 Protein
**Description:**	Mouse Polyclonal Antibody to ALPK1 Protein
**Components:**	Anti-ALPK1 Polyclonal Antibody
**Form Supplied:**	Liquid
**Storage instructions:**	Aliquot and Store at -20℃ or -80℃. Avoid Repeated Freeze / Thaw Cycles.

Sample Test Report

Samples source: protein

Western Blot Results :

ALPK1-Western Blot Result

10% SDS-PAGE
Coomassie staining

Protien marker:
GenedireX
(The BLUeye Prestained Protein Ladder)

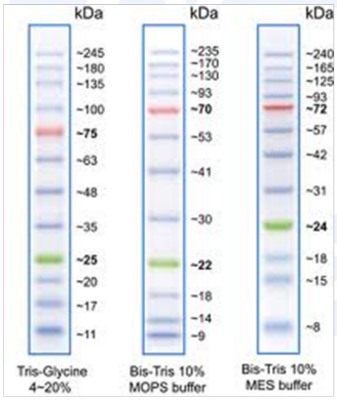

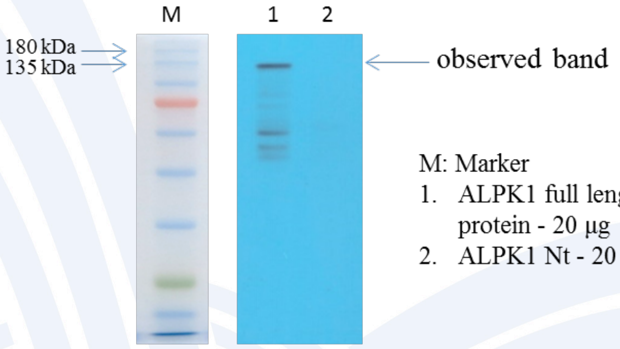

M: Marker
1. ALPK1 full length protein - 20 μg
2. ALPK1 Nt - 20 μg

Anti-ALPK1 antibody (2-1B) diluted at 1:2

Anti-mouse antibody–HRP (2nd antibody) diluted at 1:10000

- •Transfer condition(wet): 350 mA, 60 min
- •Blocking buffer: 5% skim milk in PBS
- •Blocking: 1hr (25 ℃)
- •1th antibody reaction time: overnight (4℃)
- •2nd antibody reaction time: 1hr (25 ℃)
- •Exposure Time : 5 sec

ALPK1-Western Blot Result

10% SDS-PAGE
Coomassie staining

Protien marker:
GenedireX
(The BLUeye Prestained Protein Ladder)

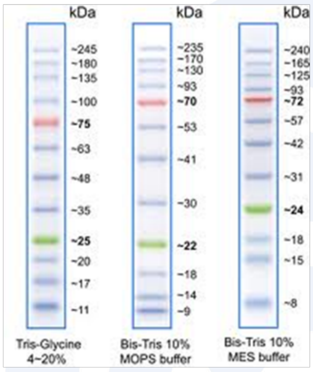

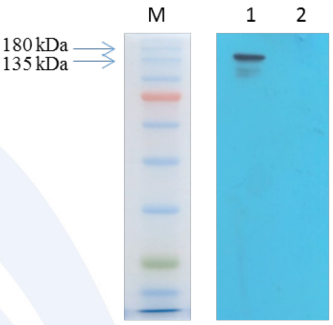

← observed band

M: Marker
1. ALPK1 full length protein - 20 µg
2. ALPK1 Nt - 20 µg

Anti-ALPK1 antibody (3-10C) diluted at 1:2

Anti-mouse antibody–HRP (2nd antibody) diluted at 1:10000

- •Transfer condition(wet): 350 mA, 60 min
- •Blocking buffer: 5% skim milk in PBS
- •Blocking: 1hr (25 ℃)
- •1th antibody reaction time: overnight (4℃)
- •2nd antibody reaction time: 1hr (25 ℃)
- •Exposure Time : 10 sec

ALPK1-Western Blot Result

10% SDS-PAGE
Coomassie staining

Protien marker:
GenedireX
(The BLUeye Prestained Protein Ladder)

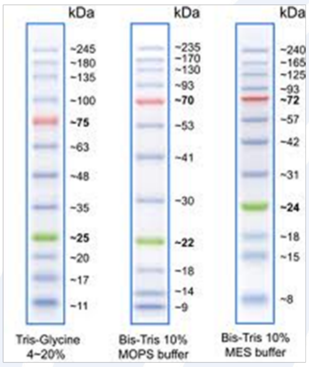

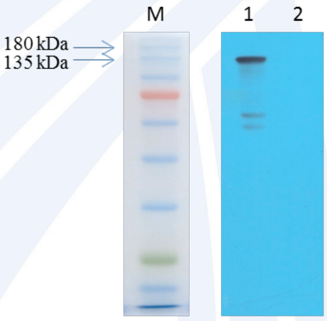

← observed band

M: Marker
1. ALPK1 full length protein - 20 µg
2. ALPK1 Nt - 20 µg

Anti-ALPK1 antibody (3-12A) diluted at 1:2

Anti-mouse antibody–HRP (2nd antibody) diluted at 1:10000

- •Transfer condition(wet): 350 mA, 60 min
- •Blocking buffer: 5% skim milk in PBS
- •Blocking: 1hr (25 ℃)
- •1th antibody reaction time: overnight (4℃)
- •2nd antibody reaction time: 1hr (25 ℃)
- •Exposure Time : 3 sec

Spectrum Analysis Report
Date: 07/15/2016 Time: 12:13
FileName: D:\Data\MALDI-TOF\20160630\ALPK1_2\0_N5\1\1SRef\pdata\1\1r

Sequence Name:		Formula:		Parentmass:	
Mass Error:		MH+ (mono):	1.008	MH+ (avg):	1.008
Threshold (a.i.):	0.000	Tolerance (Da):	0.200	Number of Peaks:	49
Above Threshold:		Assigned Peaks:		Not assigned Peaks:	

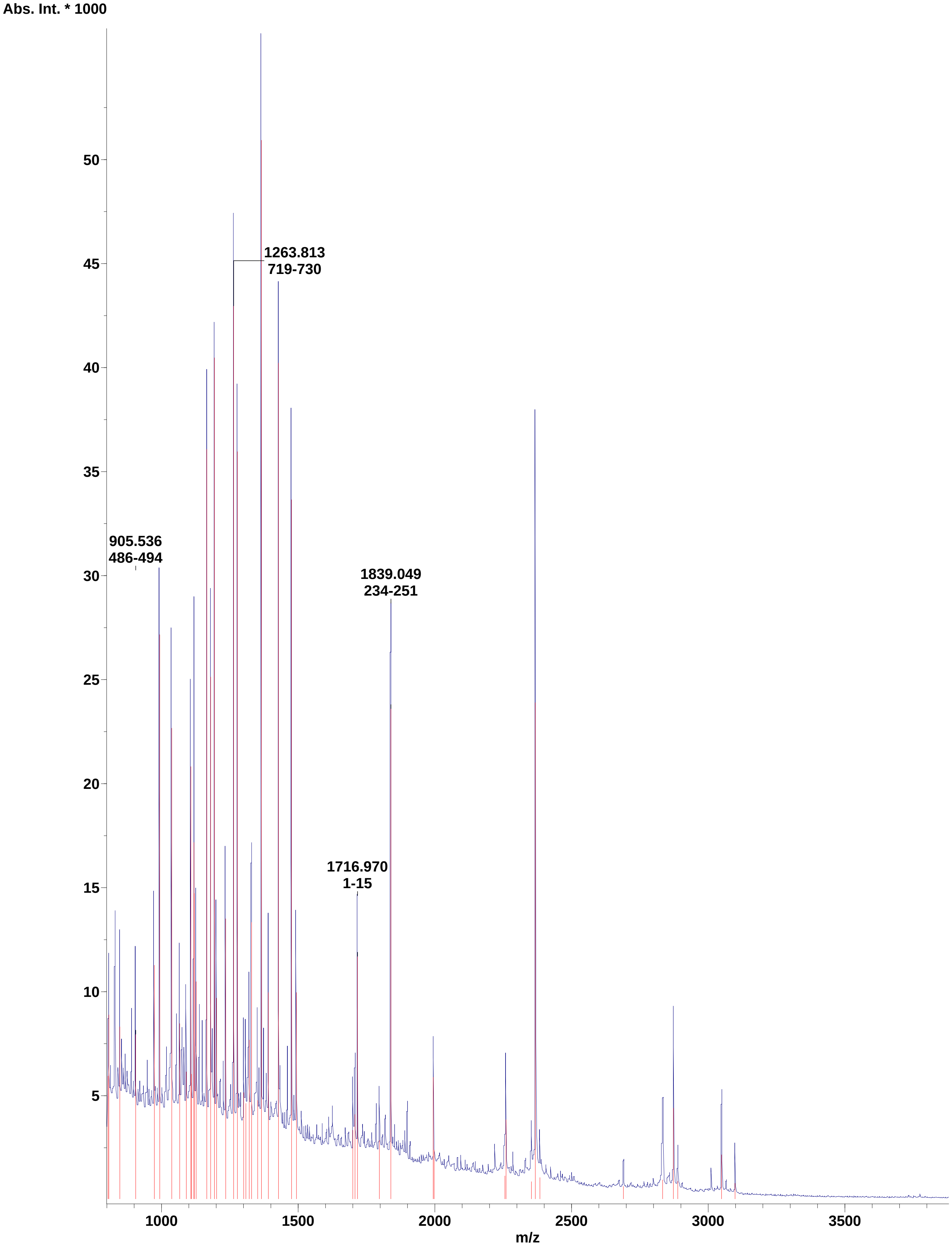

Spectrum Analysis Report
Date: 07/15/2016 Time: 12:13
FileName: D:\Data\MALDI-TOF\20160630\ALPK1_2\0_N5\1\1SRef\pdata\1\1r

Sequence data:

Intensity Coverage:	13.2 % (83470 cnts)	Sequence Coverage MS:	4.3%
Sequence Coverage MS/MS:	0.0%	pI (isoelectric point):	5.8

```
        10         20         30         40         50         60         70         80         90        100        110        120        130        140        150
MNNQKVVAVL LQECKQVLDQ LLLEAPDVSE EDKSEDQRCR ALLPSELRTL IQEAKEMKWP FVPEKWQYKQ AVGPEDKTNL KDVIGAGLQQ LLASLRASIL ARDCAAAAAI VFLVDRFLYG LDVSGKLLQV AKGLHKLQPA TPIAPQVVIR
       160        170        180        190        200        210        220        230        240        250        260        270        280        290        300
QARISVNSGK LLKAEYILSS LISNNGATGT WLYRNESDKV LVQSVCIQIR GQILQKLGMW YEAAELIWAS IVGYLALPQP DKKGLSTSLG ILADIFVSMS KNDYEKFKNN PQINLSLLKE FDHHLLSAAE ACKLAAAFSA YTPLFVLTAV
       310        320        330        340        350        360        370        380        390        400        410        420        430        440        450
NIRGTCLLSY SSSNDCPPEL KNLHLCEAKE AFEIGLLTKR DDEPVTGKQE LHSFVKAAFG LTTVHRRLHG ETGTVHAASQ LCKEAMGKLY NFSTSSRSQD REALSQEVMS VIAQVKEHLQ VQSFSNVDDR SYVPESFECR LDKLILHGQG
       460        470        480        490        500        510        520        530        540        550        560        570        580        590        600
DFQKILDTYS QHHTSVCEVF ESDCGNNKNE QKDAKTGVCI TALKTEIKNI DTVSTTQEKP HCQRDTGISS SLMGKNVQRE LRRGGRRNWT HSDAFRVSLD QDVETETEPS DYSNGEGAVF NKSLSGSQTS SAWSNLSGFS SSASWEEVNY
       610        620        630        640        650        660        670        680        690        700        710        720        730        740        750
HVDDRSARKE PGKEHLVDTQ CSTALSEELE NDREGRAMHS LHSQLHDLSL QEPNNDNLEP SQNQPQQQMP LTPFSPHNTP GIFLAPGAGL LEGAPEGIQE VRNMGPRNTS AHSRPSYRSA SWSSDSGRPK NMGTHPSVQK EEAFEIIVEF
       760        770        780        790        800        810        820        830        840        850        860        870        880        890        900
PETNCDVKDR QGKEQGEEIS ERGAGPTFKA SPSWVDPEGE TAESTEDAPL DFHRVLHNSL GNISMLPCSS FTPNWPVQNP DSRKSGGPVA EQGIDPDAST VDEEGQLLDS MDVPCTNGHG SHRLCILRQP PGQRAETPNS SVSGNILFPV
       910        920        930        940        950        960        970        980        990       1000       1010       1020       1030       1040       1050
LSEDCTTTEE GNQPGNMLNC SQNSSSSSVW WLKSPAFSSG SSEGDSPWSY LNSSGSSWVS LPGKMRKEIL EARTLQPDDF EKLLAGVRHD WLFQRLENTG VFKPSQLHRA HSALLLKYSK KSELWTAQET IVYLGDYLTV KKKGRQRNAF
      1060       1070       1080       1090       1100       1110       1120       1130       1140       1150       1160       1170       1180       1190       1200
WVHHLHQEEI LGRYVGKDYK EQKGLWHHFT DVERQMTAQH YVTEFNKRLY EQNIPTQIFY IPSTILLILE DKTIKGCISV EPYILGEFVK LSNNTKVVKT EYKATEYGLA YGHFSYEFSN HRDVVVDLQG WVTGNGKGLI YLTDPQIHSV
      1210       1220       1230       1240       1250
DQKVFTTNFG KRGIFYFFNN QHVECNEICH RLSLTRPSME KPCT
```

Display Parameter:

MH+ (mono):	1.008	MH+ (avg):	1.008	Threshold (a.i.):	0.000
Tolerance (Da):	0.200	Number of Peaks:	49		

Peaklist:

Peak	Mass	Intensity	Peak	Mass	Intensity	Peak	Mass	Intensity	Peak	Mass	Intensity
1	804.410	5763.047	2	807.459	8608.256	3	847.504	8052.646	4	905.536	7688.968
5	973.613	10921.209	6	993.595	26309.811	7	1037.622	21956.511	8	1066.623	8202.954
9	1090.613	5954.946	10	1107.632	20169.352	11	1109.593	5849.386	12	1118.589	16636.673
13	1119.646	14265.167	14	1126.617	10156.656	15	1165.695	34934.001	16	1179.705	24340.242
17	1193.718	39196.297	18	1201.708	9389.993	19	1234.782	13082.895	20	1263.813	41611.452
21	1277.829	34830.995	22	1300.679	4995.441	23	1308.758	4488.757	24	1320.714	7441.684
25	1329.768	12914.037	26	1351.765	4628.669	27	1365.763	49327.842	28	1390.802	9649.966
29	1427.912	38956.695	30	1475.882	32592.234	31	1493.860	9650.763	32	1699.908	3237.457
33	1707.893	3970.089	34	1716.970	11318.707	35	1797.047	2769.012	36	1839.049	22850.676
37	1994.016	5706.107	38	1997.194	2312.726	39	2256.177	1113.112	40	2260.354	3727.944
41	2353.137	843.469	42	2367.468	23145.138	43	2384.114	1031.795	44	2689.594	618.232
45	2833.190	925.278	46	2872.567	4261.661	47	2888.620	870.791	48	3048.744	2096.420
49	3098.140	764.047									

Copyright c 1999-2007 Bruker Daltonik GmbH
